# Laser printed two-dimensional transition metal dichalcogenides

**DOI:** 10.1038/s41598-021-81829-w

**Published:** 2021-03-04

**Authors:** Omar Adnan Abbas, Adam Henry Lewis, Nikolaos Aspiotis, Chung-Che Huang, Ioannis Zeimpekis, Daniel W. Hewak, Pier Sazio, Sakellaris Mailis

**Affiliations:** 1 Optoelectronics Research Centre, University of Southampton, Southampton, SO17 1BJ UK; 2 Present Address: Skolkovo Institute of Science and Technology, Novaya St., 100, Skolkovo, 143025 Russian Federation

**Keywords:** Materials science, Nanoscale materials, Synthesis and processing, Two-dimensional materials, Electronic devices, Nanoscience and technology, Nanoscale materials, Synthesis and processing, Two-dimensional materials

## Abstract

Laser processing is a highly versatile technique for the post-synthesis treatment and modification of transition metal dichalcogenides (TMDCs). However, to date, TMDCs synthesis typically relies on large area CVD growth and lithographic post-processing for nanodevice fabrication, thus relying heavily on complex, capital intensive, vacuum-based processing environments and fabrication tools. This inflexibility necessarily restricts the development of facile, fast, very low-cost synthesis protocols. Here we show that direct, spatially selective synthesis of 2D-TMDCs devices that exhibit excellent electrical, Raman and photoluminescence properties can be realized using laser printing under ambient conditions with minimal lithographic or thermal overheads. Our simple, elegant process can be scaled via conventional laser printing approaches including spatial light modulation and digital light engines to enable mass production protocols such as roll-to-roll processing.

## Introduction

Since the discovery of the indirect-to-direct bandgap transition in monolayer MoS_2_^1,2^, semiconducting two-dimensional transition metal dichalcogenides (2D-TMDCs) have attracted significant interest due to the plethora of interesting physical properties and their compositional tunability^3^. Properties such as bandgap tuning via thickness reduction^1,2^, high mobility and on–off ratio of 2D-TMDCs based FETs^4^ and mechanical flexibility^5^ offer a broad spectrum of prospective applications in optoelectronics^6^, photonics^6^, sensing^7^, nanoelectronics^8^, flexible and wearable electronics^9,10^.

Hence, finding suitable materials synthesis methods for TMDCs is essential for the industrial utility of these materials. Initial studies of 2D-TMDCs were performed in mechanically exfoliated, single/few layer, flakes from bulk crystals^1,2^. Mechanical exfoliation is a top down technique suitable for prototyping of devices, which however is not scalable. Liquid-phase exfoliation of TMDCs is a scalable top-down approach technique, however, thickness uniformity of liquid exfoliated TMDCs films and device performance are questionable^11^.

Despite the limited progress has been achieved in liquid exfoliation of TMDCs^12^, bottom-up synthesis approaches seem more applicable for industrial purposes. Therefore, several routes have been proposed, among them, chemical vapour deposition (CVD) has investigated extensively as this method normally produces high-quality, thickness controllable crystals^13^. However, there are still issues faced by CVD process such as mass production of wafer-scale single crystal films, high annealing temperature requirements and long processing times.

Another bottom up method is the solution-based synthesis of 2D-TMDCs which utilizes thermal decomposition of single source precursors films such as ammonium tetrathiomolybdate (NH_4_)_2_MoS_4_^14^ and ammonium tetrathiotungstate (NH_4_)_2_WS_4_^15^. Recently, roll‐to‐roll production of few-layer MoS_2_ films, tens of centimetres in length with excellent long‐range uniformity and stoichiometry have been demonstrated by this technique^16^. This promising approach could be applicable for the mass production required by industry due to cost-effectiveness and ease of growth conditions to produce large area TMDCs films. However, like CVD, this method still requires the use of a carefully controlled TMDCs growth environment, as well as standard top-down, cleanroom based lithographic post-processing for device fabrication.

Lasers have been involved in growth of 2D-TMDCs films physically via pulsed laser deposition (PLD)^17–20^ and chemically through in-situ laser sulphurisation^21^ or selenisation^22^ of transition metal oxides films. Nevertheless, the 2D-TMDCs films grown by PLD generally possess poor quality while laser sulphurised/selenised films cannot be isolated from untreated oxides precursor films and need special atmosphere for deposition. Moreover, due to high localised energy they can deliver, lasers have also been used for various post-treatments of 2D-TMDCs such as thinning^23–31^, crystallisation^32–36^, chemical modification (doping)^37–45^ and patterning^46,47^.

In this work, we present a bottom-up method for the direct synthesis and patterning of MoS_2_, WS_2_ and their heterostructures under normal room ambient operating conditions via laser-induced decomposition of (NH_4_)_2_MoS_4_ and (NH_4_)_2_WS_4_ films, which have been applied onto various substrates. This method is also capable of creating high-precision, micro-scale 2D-TMDCs heterostructures by two-step direct laser writing.

## Results and discussion

**Figure 1:**
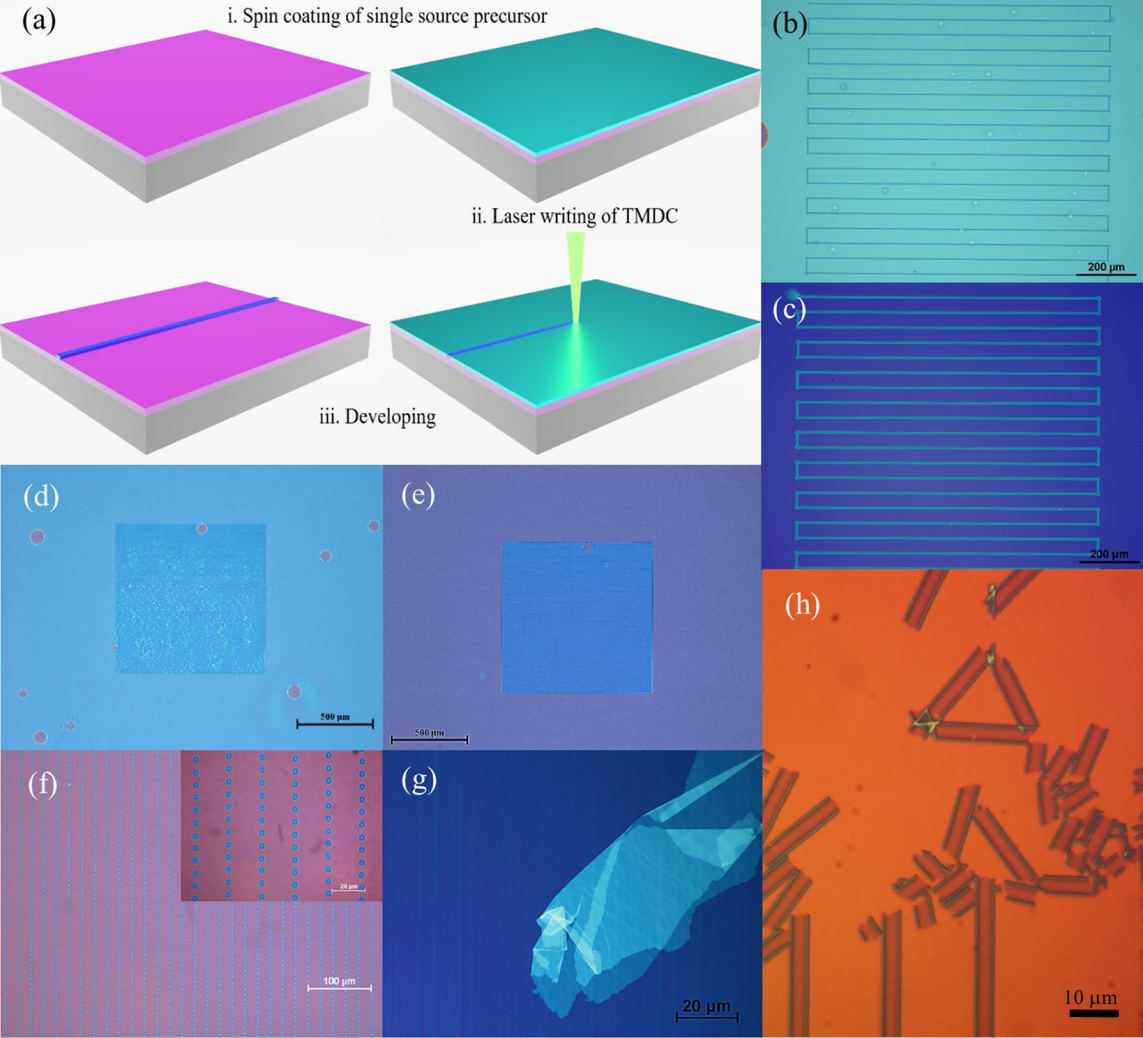
(**a**) Schematic illustration of direct laser printing of 2D-TMDCs, which comprises of: i. spin-coating of the precursor solution, ii. laser irradiation of the precursor and iii. development in organic solvent to remove unexposed precursor film. (**b**) Optical microscopy image of the laser irradiated (NH_4_)_2_MoS_4_ precursor film. The visible serpentine tracks correspond to laser synthesized MoS_2_. (**c**) Optical microscopy image of the pattern shown in (**b**) after “development” step where only the precursor film is preferentially removed leaving behind the MoS_2_ pattern. (**d**,**e**) Optical microscopy image of laser-synthesised MoS_2_ film before and after development created by overlapping raster scanning. (**f**) Optical microscopy image of WS_2_ micron-size dot array (the inset corresponds to a higher magnification image of the sample). (**g**,**h**) detached MoS_2_ film and tracks due to excessive developing time in DMF solvent.

This method can be envisioned as localised laser-printing of TMDCs. The processing steps are schematically illustrated in Fig. 1a, namely: substrate conditioning and precursor solution preparation; deposition of the precursor film by spin-coating; laser exposure to locally decompose the precursor and directly synthesise and write the TMDCs patterns and finally removal of excess unexposed precursor film in a single developing step using common organic solvents. These steps are described in detail in the “Methods” section.

The optical absorption of the single source precursors was determined from the UV–Visible transmission spectra, which were obtained from various films with different thickness/molar solution concentrations, deposited by spin coating on optically transparent silica glass substrates (see Fig. S1). This analysis revealed that the precursor films exhibit considerable absorption in the green/blue region of the visible spectrum which increases proportionally with the precursor film thickness, i.e. the concentration of the precursor solution. The optimum range for the precursor solution concentrations in terms of optical absorption and film homogeneity was identified experimentally (see Fig. S1). Taking advantage of the high absorption of the precursor in the visible spectral region, it is possible to provide the energy that is required for local thermal decomposition by irradiating the film with a focussed beam of an Argon ion laser operating in a multi-wavelength mode with dominant emission at the 488 and 514.5 nm lines.

In order to maximize the optical contrast of the laser synthesized TMDCs structures, SiO_2_/Si wafers with 285–300 nm thick oxide were the best choice of substrate for this purpose^48^. Figure 1b–f shows patterned MoS_2_ and WS_2_ that was laser-synthesised directly on SiO_2_/Si under ambient room temperature operating conditions. However, successful laser synthesis of MoS_2_ and WS_2_ were also performed on different substrates such as bulk silica glass and single crystal lithium niobate, as confirmed by Raman spectra (see Fig. S2), indicating that the method is substrate agnostic.

The serpentine tracks seen in Fig. 1b,c were produced by using a combination of two high precision Aerotech ABL1500 air bearing linear stages. Individual MoS_2_ linear tracks, or even continuous large area films can be formed using this set-up by single linear scanning or by raster scanning/overlapping of individual tracks respectively as shown in Fig. 1d,e. The laser irradiated tracks are visible under an optical microscope as shown in Fig. 1b,d as a significant optical contrast develops between the laser irradiated regions and the untreated precursor, even prior to the removal of excess precursor using DMF (dimethylformamide) solvent (see Fig. 1c,e). Similar behaviour was observed with WS_2_ and its precursor after developing in NMP (N-Methyl-2-pyrrolidone) solvent as shown in Fig. S3. An array of micron size WS_2_ islands was produced in this manner (Fig. 1f) by modulating the laser beam, using a mechanical chopper, while rastering the translation stage that supported the substrate. In the developing step, prolonged exposure of the TMDCs tracks to the DMF solvent leads to detachment and fracturing of the structures as can be seen in Fig. 1g,h due to the ease of dispersing TMDCs layers in this solvent^11^; however, using NMP as the developer solvent subsequently overcame this issue. However, Raman analysis of the laser-synthesised MoS_2_ tracks were evaluated before and after developing, suggesting that the composition of MoS_2_ was not altered by the development process, which involves an organic solvent (DMF) used as a developer as shown in Fig. S4.

Another notable feature arises from the Gaussian intensity profile of the laser beam, which produces a non-uniform temperature distribution across the laser irradiated area of the precursor films. As a result, the laser-synthesised TMDCs tracks exhibit a characteristic formation of side lobes located at the edges of the track that corresponds to wings of the laser intensity profile. This can be seen in the micrograph of Fig. 1h, where the darker edges of the side lobes are separated by ~ 4 μm, matching the laser spot size. Surface profilometry measurements of these structures confirms that their shape and height is strongly influenced by both the laser processing parameters and concentration of the precursor solution, as shown in Fig. S5a. Raman spectroscopy shows that they mainly consist of thick, poor crystallinity TMDCs “hills” (Fig. S5b) in contrast to the thinner, higher crystallinity MoS_2_ or WS_2_ central regions in-between (Fig. 1h). The ratio of the area occupied by the central regions over the edges can be tuned by changing the laser intensity, writing speed and thickness of precursor film (i.e. by changing the precursor solution concentration).

It is also important to note that exploring the parameter space of direct laser synthesis will allow thickness control of the MoS_2_ or WS_2_ via sublimation of the topmost layers^23,26^; improve the crystallinity of the TMDCs^32,33^; and affect the amount of the oxide content in the structures^37,41,42^.

**Figure 2:**
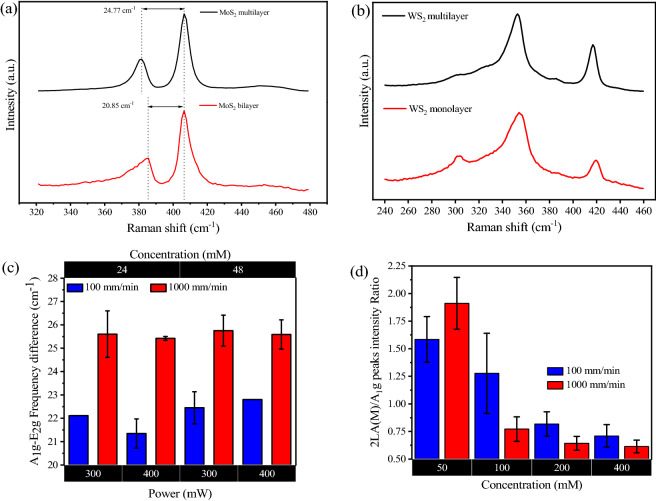
(**a**) Raman spectra corresponding to a laser synthesised bilayer (red curve) and multilayer (black curve) of MoS_2_. (**b**) Raman spectra of single layer (red curve) and multi-layer (black curve) of WS_2_. (**c**) Histogram showing the average Raman spectral separation (A_1_g-E_2_g) of MoS_2_ as function of different laser writing conditions (10 × objective used for focusing the laser beam). The error bar represents the standard deviation for four individual MoS_2_ tracks. (**d**) Graph represents the average of Raman peak intensity ratio 2LA (M) /A_1_g of WS_2_ post-deconvolution as function of different laser writing conditions. The laser power was fixed to 300 mW and 20 × objective were used to focus the laser beam. The error bars represent the standard deviation from Raman spectra obtained on five individual WS_2_ tracks.

Fig. S1 shows uniform coverage and adequate optical density of the TMDCs precursor films; this required (NH_4_)_2_MoS_4_ concentrations of 24 mM and 48 mM whereas 50, 100, 200 and 400 mM concentrations were used for (NH_4_)_2_WS_4_ spin-coating. Initially, MoS_2_ on SiO_2_/Si samples prepared with 24 mM and 48 mM (NH_4_)_2_MoS_4_ films were processed by irradiating multiple linear tracks using 300 and 400 mW power levels with a beam spot size of 3 μm, which was obtained with a 10×, 0.25 NA microscope objective. Two different stage scanning speeds, 100 and 1000 mm/min were used in these experiments. Raman spectroscopy was used to verify the formation and layer thickness of MoS_2_ from the characteristic peaks (in-plane E_2_g) and (out-of-plane A_1_g) and their spectral separation^49,50^, with the full width half maximum (FWHM) of the peaks also providing qualitative information about the crystallinity of the laser written tracks^14,16,51^. Raman spectra corresponding to MoS_2_ films are shown in Fig. 2a. Bilayer formation was observed using the 24 mM precursor solution at 400 mW laser power and 100 mm/min scanning speed. From Fig. 2c, which shows the spectral separation of the two characteristic Raman peaks for MoS_2_, it can be deduced that MoS_2_ films synthesised using a scanning speed of 100 mm/min are thinner as compared to the ones synthesised with 1000 mm/min for the same laser power. An overview of the Raman results, however, indicates that the number of MoS_2_ layers depends on both laser intensity and laser dwell time, which is controlled by the speed of scanning. Further Raman analysis, shown in Fig. S6c, confirms that the spectral separation between the well-pronounced in-plane (E_2_g) and out-of-plane (A_1_g) peaks is smaller for the slower writing speed, indicating thinner MoS_2_ layer. Notably, extended Raman spectra (up to 900 cm^−1^) on the same MoS_2_ tracks, shown in Fig. S6b, did not indicate formation of molybdenum trioxide (molybdenum trioxide peaks are located at 666 cm^−1^ and 820 cm^−1^)^52^ irrespective of synthesis in oxygen-rich, ambient conditions.

The impact of the laser writing speed on the thickness of the resulting MoS_2_ can be explained as follow; the local temperature of the irradiated precursor at the focal point is regulated by the laser intensity. This intensity can be controlled by the laser power and beam spot size at focus (Intensity I = P/A where P is the power and A is the irradiated area)^53^. The laser induced temperature change is much faster compared with practical laser scanning speeds and so the precursor temperature dynamics reach steady state before the beam has the chance to move significantly. Therefore, the scanning speed controls the dwell time of the beam, in other words the duration of steady state heating. Consequently, the laser energy fluence (I × t, where t is the dwell time of the laser beam) delivered to the TMDCs precursors films will be higher for the films written by slower writing speed (longer interaction time) compared to faster speed (shorter interaction time)^53^. Longer irradiation times can result in the thinning of the synthesised MoS_2_ as have been demonstrated in the literature^23^.

Consistent formation of WS_2_ on SiO_2_/Si substrates (using 50, 100, 200 and 400 mM concentrations of (NH_4_)_2_WS_4_ to create precursor films) was obtained for 300 mW incident laser power and a laser spot size of 1.2 μm which was formed by a 20×, 0.4 NA microscope objective, at two writing speeds; 100 and 1000 mm/min. The WS_2_ precursor films has lower absorption for visible light in comparison to the MoS_2_ precursor films (Fig S1). This means for the same irradiation conditions the local temperature of the WS_2_ precursor film will be lower as compared to the MoS_2_ precursor films. For this reason, tighter focusing using a 20× objective lens was utilised to increase the laser intensity at the irradiated spot.

As shown in Fig. S7a,b, the out-of-plane Raman mode A_1_g of WS_2_ (around 420 cm^−1^) was pronounced for all growth conditions. The Raman peak at around 350 cm^−1^ is a convolved peak that includes in-plane Raman mode E_2_g and longitudinal acoustic mode 2LA (M), which arises due to the resonance with excitation wavelength^54^. The contribution of the 2LA (M) peak was obtained by deconvolution of the composite peak, as shown in Fig. S7c.

Figure S7c shows an example of laser written WS_2_ Raman spectrum where the intensity of 2LA(M) Raman peak is lower than E^1^_2g_ peak intensity. This peak ratio agrees with our previous report in few-layer WS_2_ films grown by thermal decomposition of the same precursor at 500 °C^15^. However, this ratio is reversed (E^1^_2g_ peak intensity becomes lower than 2LA(M)) when the WS_2_ films are annealed to a higher temperature (1000 °C)^15^. Moreover, the peak position of 2LA(M) is red shifted to 345 cm^−1^ compared to previous reports^15,54^ by 4–5 cm^−1^. Similar red shift of the 2LA(M) peak position has been previously observed in vertically oriented WS_2_ nanosheets^55^. The red shift in our laser synthesised material could be an indication of strain.

For WS_2_, the 2LA(M)/A_1_g peak intensity ratio provides information about the number of layers in the film where a higher ratio corresponds to a fewer number of layers^54^. Figure 2b shows WS_2_ Raman spectra corresponding to single layer and multilayer laser-synthesised films. For WS_2_ formed using a precursor solution concentration of 50 mM, which is the lowest concentration used in our experiments, the 2LA (M)/A_1_g peak intensity ratio becomes 1.9, which correspond to single layer^54^ WS_2_. Figure 2d shows a histogram of the 2LA (M)/A_1_g peak intensity ratio as a function of growth conditions. The gradual decrease in peak intensity ratio with increasing precursor film concentration means that the thickness of laser-written WS_2_ increases proportionally. However, no significant changes in spectral separation of the WS_2_ peaks corresponding to layer number changes were observed (Fig. S7d), possibly due to the low sensitivity of tungsten atoms to vibrational modes changes^56^.

Importantly, similar to the case of laser-synthesised MoS_2_, despite the oxygen-rich ambient conditions associated with our direct laser synthesis and patterning method, there was no indication of significant tungsten oxide formation. As shown in Fig. S7b, a weak and broad peak appears near 700 cm^−1^, whereas the Raman characteristics peaks of WO_3_ are usually located at 710 cm^−1^ and 810 cm^−1^^57,58^.

The full width half maximum (FWHM) of Raman peaks can provide an indication of the crystallinity of the synthesised MoS_2_ and WS_2_ films^59^. The literature suggests that the crystallinity of the deposited film is affected by the annealing temperature therefore it is expected that the crystallinity of our laser synthesised films will be affected by the laser irradiation conditions. Although we did not have the means for accurate measurement of the local temperature at the irradiated spot, it is possible to assign a synthesis temperature by comparing the FWHM values that we observed to the ones found in the literature^14,16,51,60^. MoS_2_ films grown by thermal decomposition at 500 °C exhibited FWHM values of 10 and 9 cm^−1^ for E^1^_2g_ and A_1g_ peaks respectively^14^. Further annealing at 1000 °C reduced the FWHM to 6 cm^−1^ for both peaks^14^. In our laser synthesised MoS_2_, the minimum FWHM was around 6.75 and 5.75 cm^−1^. This indicates that the synthesis temperature for laser synthesised MoS_2_ was close to 1000 °C.

We have provided statistical analysis for FWHM of Raman peaks for both MoS_2_ (Fig S6d) and WS_2_ (Fig S7e,f) synthesised at different irradiation conditions, which can be used to estimate the annealing temperature for each condition. The FWHM of A_1_g for WS_2_ was seen to decrease as a function of the precursor concentration indicating the crystallinity is improved for thicker starting precursor films (Fig. S7e).

Photoluminescence (PL) spectroscopy was also employed for probing the number of layers of laser written TMDCs films^1,2,61,62^. The same proprietary Raman spectroscopy system was used to obtain room-temperature PL spectra from our WS_2_ films, which are shown in Fig. S8. The spectra exhibit a clear concentration-dependent blue shift of the PL peak as the concentration of the precursor films spun-coated on the substrate is reduced. The PL peak appears at 1.987 eV (624 nm wavelength) for the highest precursor concentration of 400 mM and shifts to 2 eV (620 nm wavelength) for 50 mM using the same laser irradiation conditions. However, no PL was observed in MoS_2_ films for all laser writing conditions and precursor concentrations, possibly due to low quantum yield of MoS_2_ compared to WS_2_^61^.

Surface (stylus) profilometry was also performed on MoS_2_ and WS_2_ tracks to assess the film thickness. The profilometry measurements, which are summarized in the histograms shown in Fig. S9, are in good agreement with the Raman and PL spectroscopy analysis.

We note that numerous reports in the literature amply demonstrate that real-time, in-situ Raman spectroscopy is a highly versatile, general purpose diagnostic tool for 2D materials which can be used to analyse for example, the dynamics of laser thinning of MoS_2_ flakes as well as laser crystallization kinetics for amorphous MoS_2_ films deposited by sputtering^29,33^. Serendipitously, we have also discovered that micro-probe analysis (Renishaw InVia Raman Microscope operated at 532 nm with a 50 × 0.75NA microscope objective) of our spin-coated single-source precursor films resulted in the simultaneous laser synthesis and Raman characterisation of TMDCs layers.

Power levels of 3, 22 and 40 mW were investigated with static exposure on 48 mM precursor concentration films. At low power, no MoS_2_ or WS_2_ peaks were observed. Significantly, at medium power, characteristic in-plane (380.3 cm^−1^, E_2_g) and out-of-plane (402.5 cm^−1^, A_1_g) peaks became pronounced, revealing rapid formation of MoS_2_. At the higher end of available pump laser power in our Raman spectroscopy setup, the same Raman peaks of MoS_2_ (380.3 cm^−1^, 402.5 cm^−1^) and WS_2_ (convoluted peak at 351.6 cm^−1^ and out-of-plane peak 415.6 cm^−1^) emerged using both single-source precursors films, as can be seen in Fig. S10.

Despite this successful demonstration, it should be noted that the performance parameters of the proprietary Raman laser system are necessarily limited by design, especially in terms of maximum power (40 mW). As such, this particular system is not flexible enough to study the effect of laser power on thickness, crystallinity and oxide doping of the TMDCs films and therefore our customised, more powerful and versatile laser writing setup is essential to study thoroughly these parameters.

Further insight into the exact composition and stoichiometry as a function of the growth conditions of our films was provided by X-ray photoelectron spectroscopy (XPS). XPS investigation was performed on large area laser-synthesised MoS_2_ and WS_2_ using various precursor concentrations. Films with an area of 1 mm^2^, which is a large enough area for the system to collect accurate data for our specific analysis, were produced by overlapping raster scanning as in Fig. 1d,e. The XPS spectra, which were obtained from these films are shown in Fig. S11 and Fig. S12.

The second Mo ^(6+)^ and third Mo ^(5+)^ doublets, which can be observed in Fig. S11 correspond to molybdenum oxide. These oxide species were not detected by Raman spectroscopy, which indicates they are substitution for sulphur vacancies in the MoS_2_ lattice rather than individual molybdenum oxides layers^34^. The second doublet W^(6+)^ f_7/2_ and W^(6+)^ f_5/2_ in W core level of Fig. S12 corresponds to WO_3_ formation and the binding energies for this doublet is identical to that reported previously for WO_3_^63,64^. However, Raman spectroscopy (Fig. S7b) again did not reveal any obvious vibrational modes for the oxide. This indicates that, just as with the MoS_2_ layers, the films contain oxidised WS_2_ rather than two individual layers of WS_2_ and WO_3_ (but with a higher level of oxidation for WS_2_ compared to MoS_2_)^34^. We hypothesize that the oxide content in both MoS_2_ and WS_2_ films has been significantly increased compared to individual tracks because the large area, overlapping raster scanned films which are required for the XPS analysis subjected the material to prolonged laser exposure which can be considered as the equivalent of prolonged thermal annealing in an oxidizing atmosphere.

The collective results of XPS analysis, PL spectroscopy and stylus profilometry indicate that precursors films, which are prepared at lower concentrations, result in thinner TMDCs films with lower oxide content. However, the Raman spectroscopy of these thinner films showed broader Raman resonances, suggesting compromised crystallinity.

**Figure 3:**
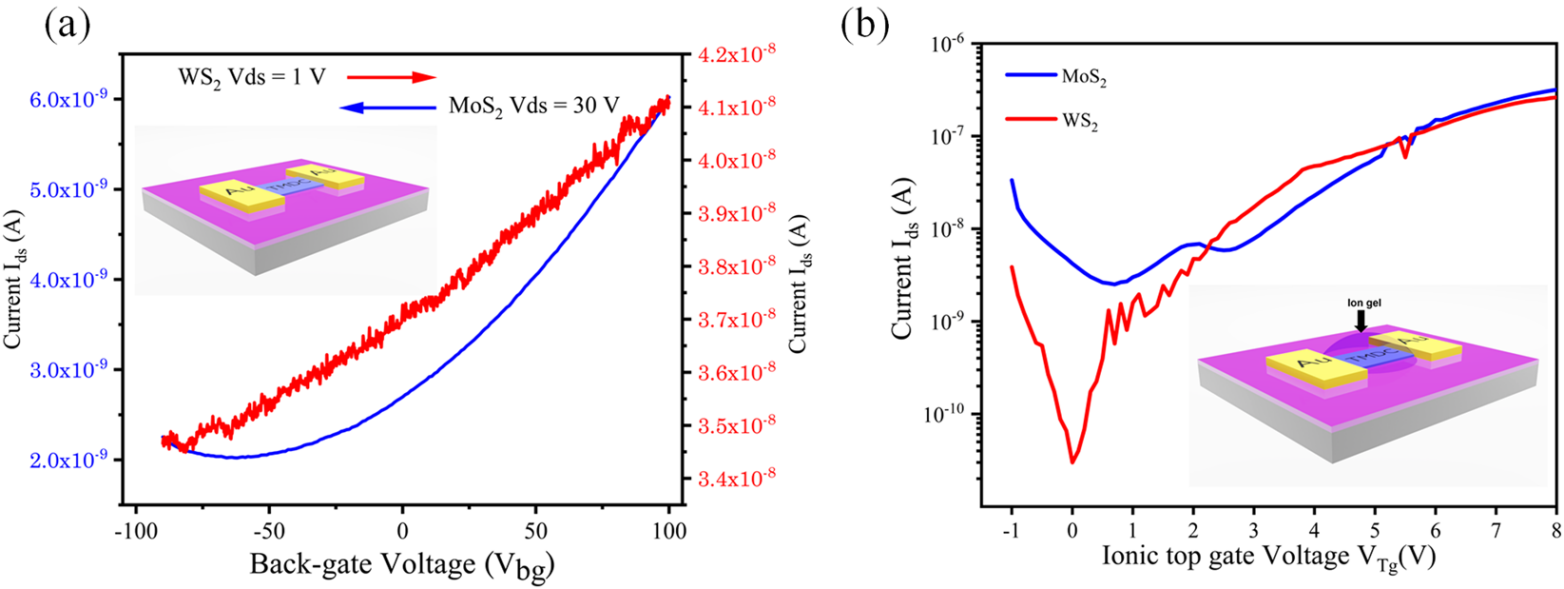
Transfer characteristics of laser-synthesised TMDCs-FET with (**a**) back-gated configuration and (**b**) Ionic top-gated configuration. Device schematics are also shown in inset images.

Finally, potential electronic device applications of our laser-synthesized TMDCs were explored using field effect transistor (FET) arrangements. We used two configurations: back gate, with the conductive silicon substrate as the gate electrode and an ionic top gate configuration for both MoS_2_ and WS_2_. Back-gated devices show modest performance in terms of both mobility (5.5 × 10^–5^ cm^2^/V. s for MoS_2_ and 1.1 × 10^–4^ cm^2^/V. s for WS_2_ calculated using the equation in Fig. S13) and on–off ratio (3 for MoS_2_ and 1.2 for WS_2_). Figure 3a shows the transfer characteristics of back gated MoS_2_ and WS_2_ FETs with device schematic. The low mobility and on–off ratio that we observed are similar to other TMDCs devices reported in the literature that were also fabricated using single source precursors, but where the TMDCs channels were patterned by conventional top-down lithography^14,15^. However, when the ionic top-gate FET configuration was used, the values for both the mobility and on–off ratio improved significantly due to the higher capacitance of the ionic gate and the encapsulation of the TMDCs channels. Figure 3b shows the transfer characteristics of ionic top gated MoS_2_ and WS_2_ FETs and the device schematic. The mobility extracted for MoS_2_ is 7 × 10^–3^ cm^2^/V. s and 6 × 10^–3^ cm^2^/V.s for the WS_2_ device, while the on–off ratio for the MoS_2_ FET is > 10^2^ and ~ 10^4^ for WS_2_ which present orders of magnitude improvement over the back gated configuration.

Conventional vertical heterostructure semiconductor growth technologies have enabled revolutionary device designs, from modulation doped high mobility electron transistors, through to quantum cascade lasers. Given the plethora of available compositions and combinations, it is strongly anticipated that similar technological progress can be realised with TMDCs once a large-scale fabrication technique can be developed that allows for the facile assembly of 2D layers, whilst preserving sharp interfaces. Our direct laser writing approach offers the opportunity for heterostructuring of TMDCs by sequential laser synthesis of individual precursor layers to create single and multiple-overlapped tracks and arrays of WS_2_/MoS_2_ interfaces. Strong interlayer excitonic transitions in such type-II heterostructure systems can generate intrinsic p–n junctions^65^, ubiquitous in incumbent microelectronic devices.

**Figure 4:**
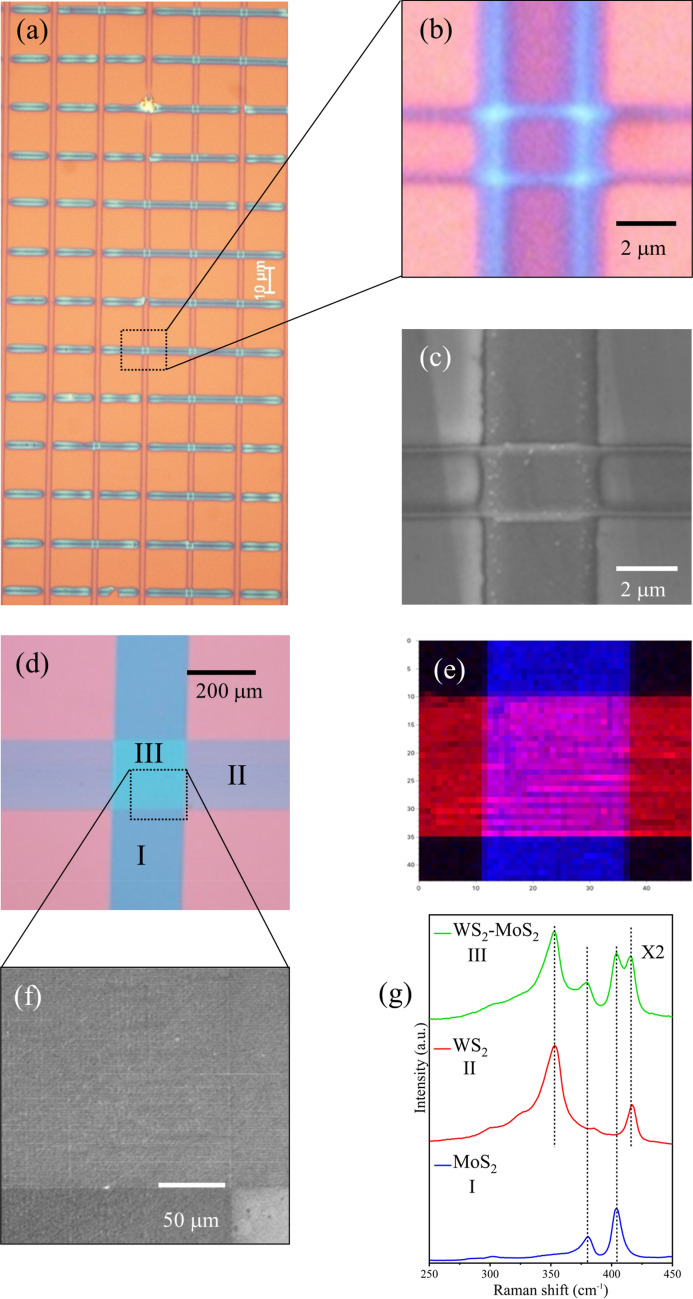
(**a**) Optical microscopy image of a WS_2_/MoS_2_ heterostructure array. (**b**,**c**) Optical microscopy image and Scanning Electron microscopy (SEM) of a single WS_2_/MoS_2_ heterostructure. (**d**) Optical microscopy image of an array of multiple overlapping WS_2_/MoS_2_ tracks forming a large area heterostructure. (**e**) False colour X-Ray fluorescence map of the large area WS_2_/MoS_2_ heterostructure shown in (**d**), where the red and blue bands correspond to X-Ray fluorescence from Mo and W atoms respectively. (**f**) Scanning Electron microscopy (SEM) of (**d**) showing crosshatch pattern. (**g**) Raman analysis of (**d**) is strongly indicative of a sharp vertical interface with the characteristic modes of both materials (I = MoS_2_, II = WS_2_) present in region III.

Optical and scanning electron microscopy images of various WS_2_/MoS_2_ heterostructures are presented in Fig. 4. The array shown in Fig. 4a is formed by synthesis of a set of WS_2_ tracks separated by 20 μm followed by synthesis of a set of MoS_2_ tracks, rotated by 90 degrees with respect to the WS_2_ tracks, to form a cross-hatch pattern. The unused precursor was removed in intermediate processing steps by development in NMP solvent. The heterostructures are formed at the overlapping points between the two orthogonal sets of tracks. Figure 4b,c shows detail of a single MoS_2_/WS_2_ heterostructure using optical microscopy and SEM at the same resolution. Although numerous junctions were created successfully, as can be seen in Fig. 4a and Fig. S14, some are damaged due to the non-optimised synthesis parameters. Raster scanned, overlapping MoS_2_/WS_2_ heterostructure are also shown as optical and SEM micrographs in Fig. 4d,f and at higher optical resolution in Fig. S14, where the individual lines forming the large area junction create a “tartan” pattern.

To verify the continuity of the elemental content in the patterned heterostructure we gained access to the micro-XRD beam line (I18) at Diamond Light source in Harwell campus Oxfordshire. The beamline uses a focussed X-Ray beam, with tunable energy, that was raster-scanned on the sample while simultaneously acquiring fluorescence spectra to create X-Ray fluorescence (XRF) maps of the MoS_2_/WS_2_ heterostructure. Figure 4e, shows such an XRF map where the Mo-Kα and W-Lα emission as registered in false colour (red for Mo-Kα and blue for W-Lα). The X-Ray emission of each element is spatially distinct in the areas outside the overlapping region and mixed on the heterostructure as expected. Additional evidence of the material state on the heterostructure was provided by Raman analysis of this area, as shown in Fig. 4g, where the characteristic modes of both materials are present in the heterostructure region. A large, dense (and potentially addressable) array of 50 × 50 MoS_2_/WS_2_ tracks and (approx. 2500) heterostructures was also imaged in false colour using the XRF facility as shown in Fig. S15. The elemental spatial distribution is again well defined, despite the close packing of the lines (50 lines/mm).

## Conclusion

We have demonstrated a bottom-up, spatially selective laser synthesis approach that utilizes solution-based single source precursors to create patterned MoS_2_, WS_2_ films and their heterostructures. This method can form microscale structures with thickness down to monolayer for WS_2_ and bilayers for MoS_2_. X-ray photoelectron spectroscopy results suggest that instant decomposition of ammonium tetrathiomolybdate (NH_4_)_2_MoS_4_ and ammonium tetrathiotungstate (NH_4_)_2_WS_4_ can be achieved to create MoS_2_ and WS_2_ respectively even under ambient, strongly oxidizing conditions when the precursor films are exposed to localised high energy of visible CW lasers. Thus, unlike TMDCs films grown by global pyrolysis of these precursors, the controlled growth environments such as low pressures and inert gases are eliminated which makes this production methodology much simpler. Additionally, due to localised decomposition which enables low-temperature processing, TMDCs structures have been successfully deposited on different types of substrates. Raman and PL spectroscopy revealed that thickness, crystallinity and oxide content of 2D-TMDCs can be tuned solely via precursor film thickness and laser writing parameters (intensity and writing speed). Field effect transistors (FETs) have been fabricated and characterised to show the potential electronic applications of 2D-TMDCs laser written structures. High-precision MoS_2_/WS_2_ heterostructures with lateral dimensions reaching the diffraction limit of the laser have been realised using this method by sequential laser synthesis of MoS_2_ and WS_2_ precursors.

In prospective, our laser printing process for the formation of 2D TMDCs device designs and heterostructures provides an elegant, flexible and scalable digital additive manufacturing capability. Room temperature synthesis and direct writing under ambient conditions is clearly compatible with future industrial processes in which numerous TMDCs materials could be patterned *in-situ* and used to augment the functionality of incumbent, bulk, wafer-based semiconductor technologies. Furthermore, fully integrated, instantaneous spectroscopic data and diagnostics including Raman, PL and pyrometry of the synthesis patterning will allow *in-situ* optimisation of the printed 2D materials by adjusting irradiation parameters in real time (e.g. laser fluence, duty cycles, CW or even ultrafast laser processing using nonlinear two-photon absorption for potential sub-micron patterning resolution beyond the Abbe limit). Adding these characterisation tools will not only result in a significant saving in offline analysis, but also allow for future efficient exploration of much wider parameter space than that detailed here, via the rational implementation of machine learning protocols to enable full control over the quality and number of printed TMDCs layers. Laser sublimation and thinning of layers combined with annealing and iterative processing can also improve the crystallinity as well as allowing for bandgap engineering, alloy and heterostructure formation to enable the rapid discovery of radical electronic and photonic device designs.

## Methods

### Substrates and precursor solution preparation

In order to ensure a high level of continuity and uniformity of laser printed TMDCs structures in terms of thickness and quality, the precursor film preparation is a critical step which must be highly reproducible. Therefore, the hydrophilicity of the substrates and the solubility of the precursors in their solutions have been optimised in the following manner:

#### Substrate preparation

Heavily doped n-type silicon substrates (SiO_2_/Si with oxide thickness 285–300 nm) were cleaved to square sizes (approx. 1 and 1.5 cm^2^). After cleaving, the substrates were cleaned sequentially by acetone, isopropanol, methanol and deionized water then dried by nitrogen gun. To promote the adhesion between the MoS_2_ precursor films and the substrates, Yang et al. used oxygen plasma ashing for a few seconds at low power^51^. However, we found this to be inadequate and instead, if the substrates are oxygen plasma treated prior to the spin coating process for 10–15 min at 0.1 mbar pressure (oxygen flow 1000 mL/min) and 1000 W microwave power, the wettability of the (MoS_2_ and WS_2_) precursor solutions will be significantly enhanced on the surface of the SiO_2_/Si substrates.

#### Preparation of precursor solutions

The initial concentration of the precursor solutions that are spin coated on the substrates play an important role on the final properties of the TMDCs structure. Therefore, the maximum concentrations have been chosen carefully to ensure optimum coverage and uniformity of precursor films after spin-coating whilst maintaining adequate optical density. In addition, this range of concentrations also ensured that unwanted clustering and film inhomogeneity were avoided. These concentrations are 24 mM and 48 mM for ammonium tetrathiomolybdate (NH_4_)_2_MoS_4_ while the concentrations used for ammonium tetrathiotungstate (NH_4_)_2_WS_4_ were 50, 100, 200 and 400 mM due to lower absorption in the visible spectral range of this precursor (see Fig. S1). The solutions of TMDCs precursors were prepared as follow:

i. MoS_2_ precursor preparation:
  Batches of 31 mg and 62 mg of ammonium tetrathiomolybdate (NH_4_)_2_MoS_4_ were dissolved in 5 mL of solvent system which contains: 2 mL DMF, 2 mL n-butylamine and 1 mL 2-aminoethanol to create 24 mM and 48 mM concentrations of precursor solution. The solutions were sonicated for 15–20 min to improve the solubility and homogeneity. The solvent system was adopted with trivial modification from Yang et al.^51^ .
ii. WS_2_ precursor preparation:
  Batches of 105, 209, 418 and 835 mg of ammonium tetrathiotungstate (NH_4_)_2_WS_4_ were dissolved in 6 mL of solvent system which contains: 3 mL NMP, 2 mL n-butylamine and 1 mL 2-aminoethanol to create 50, 100, 200 and 400 mM concentrations of precursor solution. Solutions were sonicated for 1 h at 70 °C to improve the solubility and homogeneity. The solvent system was adopted from our previous work, Abbas et al.^15^.

### Spin coating

The solutions were spin coated using each precursor for 1 min with the following details: Step 1—ramp 5 s, dwell time 5 s, rpm 500; Step 2—ramp 5 s, dwell time 45 s, 3000 rpm for MoS_2_ precursor, 6000 for WS_2_ precursor. After spinning, the samples were prebaked for 1 to 5 min to evaporate the solvents at 90 °C for MoS_2_ precursor and 140 °C for WS_2_ precursor.

### Laser writing parameters

The laser radiation source was a C.W. Ar^+^ laser (Coherent Innova 90C) operating in multi wavelength mode (predominantly λ = 514.5 nm). Laser powers in the range of 300 to 400 mW were used for the synthesis of MoS_2_ typically using a 10×, 0.25 NA objective (laser intensities in the range of 3.1 ± 0.1 MW/cm^2^ to 6.4 ± 0.1 MW/cm^2^), whereas WS_2_ was synthesised using 300mW of laser power and 20×, 0.4 NA microscope objective (laser intensity 7.5 ± 0.1 MW/cm^2^). The laser scanning speed that resulted in successful synthesis of linear tracks was in the range 100–1000 mm/min.

### Developing the samples in solvents after laser writing

After laser exposure, the samples were immersed in solvents, using DMF for MoS_2_ or NMP for either MoS_2_ or WS_2_ for 60 s with gentle agitation to make sure that the untreated precursor films are totally removed (requiring multiple immersions in the solvent especially with high concentrations). After developing, the samples were dried by nitrogen gun. It is generally preferable to use NMP rather DMF for developing as the latter could cause partial lift-off of the deposited TMDCs film, as can be seen in Fig. 1g,h.

### Characterisation

The transmission spectra of the precursor films were obtained using a Jasco 570 UV/Vis spectrophotometer. Raman and photoluminescence spectroscopy were performed using a Invia Raman Microscope (Renishaw) system with a 532 nm excitation wavelength at 20 mW power (unless otherwise stated) and 50 × objective. Stylus profilometry was performed using KLA Tencor P-16 Stylus Profiler. XPS spectroscopy was obtained using a Thermo Fisher Scientific Thetaprobe system. SEM images of the TMDCs heterostructures were collected using a Thermo Scientific Quattro ESEM. XRF spectroscopy mapping was obtained using the micro-XRD beam line I18 at the Diamond Light Source, Harwell campus, Oxfordshire, UK. Electrical measurements were performed in ambient conditions using an Agilent 4155C semiconductor parameter analyser connected to a cascade micropositioning stage.

### Device fabrication

#### Back-gated devices

The MoS_2_ and WS_2_ FET devices were directly laser printed onto SiO_2_/Si chips without the need for transfer from other substrates. The width of the channels (200 μm for MoS_2_ and 250 μm for WS_2_) were defined by overlapping raster scanning with the following parameters: solution concentration of precursor film: 48 for MoS_2_ and 100 mM for WS_2_; Power (intensity): 300 mW (10 × objective) for MoS_2_ and 250 mW (20 × objective) for WS_2_; writing speed 100 mm/min for both; spacing 2 μm. After laser writing, the MoS_2_ film was developed in DMF and WS_2_ was developed in NMP to remove the untreated areas of the precursor film and form the FET channel. Source-drain ohmic contacts were fabricated using standard photolithographic protocols for metal lift-off. Subsequent e-beam evaporation was then employed with 10 nm In/100 nm Au and channel length 100 μm for MoS_2_ FET and 10 μm for WS_2_ device. For photolithography, S1805 were spun coated over the samples for 1 min at 5000 rpm and soft baked for 1 min at 110 °C. After UV exposure, the samples were developed in MF-319 developer for 5–6 s followed by rinsing in de-ionized water. It is worth mentioning that S1805 was used to avoid longer developing time in MF-319 which start damaging the MoS_2_ films when the developing time exceeds 10–20 s.

#### Ionic gel top-gated devices

The MoS_2_ and WS_2_ FET devices were directly laser printed onto SiO_2_/Si chips without the need for transfer from other substrates. The width of the channels (250 μm for both) was defined by raster scanning with following parameters: solution concentration of precursor film: 48 mM for MoS_2_ and 100 mM for WS_2_; Power (intensity): 300 mW (10 × objective) for MoS_2_ and 250 mW (20 × objective) for WS_2_; writing speed 100 mm/min for both; spacing 2 μm. After laser writing, the MoS_2_ film was developed in DMF and WS_2_ was developed in NMP to remove the untreated areas of the precursor film and form the FET channel. Source-drain ohmic contacts were fabricated using standard photolithographic protocols for metal lift-off. Subsequent e-beam evaporation was then employed with 10 nm In/50 nm Au and channel length 10 μm for both devices.

The ionic-gel gate was prepared by mixing lithium perchlorate (LiClO_4_) and polyethylene oxide (PEO) with mass ratio (1 g to 0.12 g) and dissolving them in 40 mL of methanol. The solution was heated at 50 °C until it became viscous then drop casted over the FET devices and left for one hour to dry and solidify before the measurements were undertaken. The ionic gel recipe and preparation has been adopted with trivial modifications from Hu et al.^66^.

### Heterostructures

Single and multi-track MoS_2_/WS_2_ heterostructures were laser written using the following steps: the substrates were cleaned and plasma conditioned prior to spin-coating of the WS_2_ precursor solution with 100 mM concentration. Subsequently, the WS_2_ structures were synthesised using laser power at 250 mW, 20 × objective and 100 mm/min writing speed. Next, the WS_2_ sample was developed in NMP to remove the untreated precursor film. Afterwards, the MoS_2_ precursor solution with concentration 48 mM was spun-coated directly on the WS_2_ laser-processed sample and the MoS_2_ lines written perpendicularly to WS_2_ tracks using laser power 200 mW, 20 × objective and 100 mm/min writing speed. Finally, the sample was immersed again in NMP to develop the MoS_2_/WS_2_ heterostructures. The reason behind this sequence of precursor film deposition is that laser formation of WS_2_ essentially requires higher laser intensity which might be damaging to MoS_2_ structures if they were written first.

The observed damage to the WS_2_ tracks in Fig. 4a and Fig. S14 after the second developing step when the MoS_2_ precursor film was cast, is most likely due to the MoS_2_ precursor solution containing DMF which could partially dissolve the WS_2_ structures. Thus, further optimisation is needed for double-layer deposition of precursor films and for the laser writing conditions to create large, dense and addressable heterostructure arrays.

## Supplementary Information

Supplementary Information

## Data Availability

All experimental data required to support the findings of this work are presented either in the main article or the supplementary information.
