# Supplementary material for: Laser printed two-dimensional transition metal dichalcogenides
Source: Sci Rep. 2021 Mar 4;11:5211. doi: 10.1038/s41598-021-81829-w (PMC7933426; doi:10.1038/s41598-021-81829-w)
Supplement: Supplementary file 1 — Supplementary Information [file 41598_2021_81829_MOESM1_ESM.pdf]

Supplementary Information

Laser Printed Two-Dimensional Transition Metal Dichalcogenides

Omar Adnan Abbas[1], Adam Henry Lewis[1], Nikolaos Aspiotis[1], Chung-Che Huang[1], Ioannis Zeimpekis[1], Daniel W Hewak[1], Pier Sazio[1] and Sakellaris Mailis[1,2]*

1. Optoelectronics Research Centre, University of Southampton, Southampton, SO17 1BJ, United Kingdom

2. Current address: Skolkovo Institute of Science and Technology Novaya St., 100, Skolkovo 143025, Russian Federation

Corresponding Author: Sakellaris Mailis

* E-mail: s.mailis@skoltech.ru

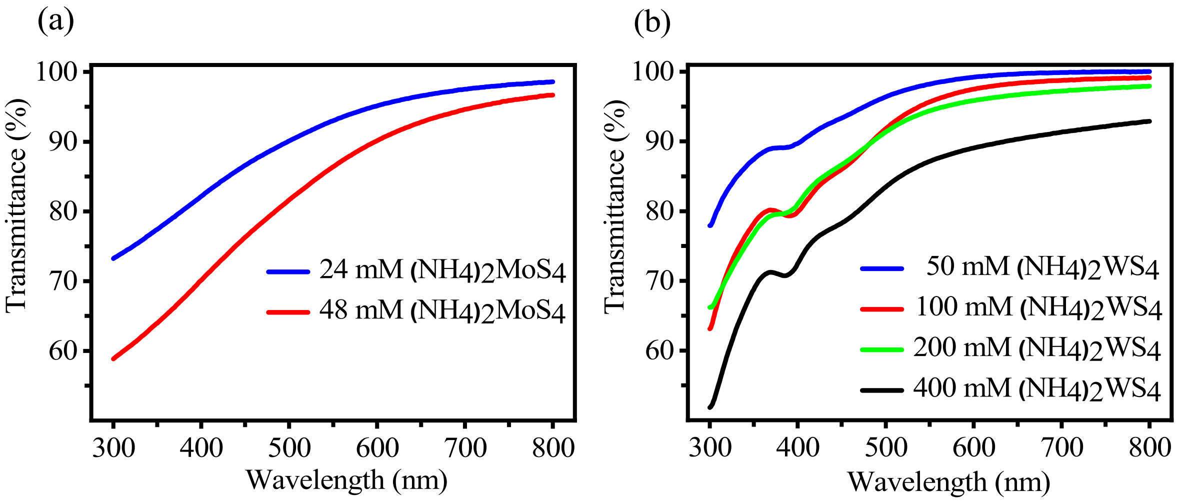

Figure S1: UV-VIS transmission spectra of (a) ammonium tetrathiomolybdate $(NH_4)_2MoS_4$ with 24 & 48 mM films deposited on silica substrates and (b) ammonium tetrathiotungstate $(NH_4)_2WS_4$ with 50, 100, 200 and 400 mM films deposited on silica substrates. The absorption of the precursor films in the blue-green region of the visible spectrum increases with concentration of the precursor solution. Note that for the ammonium tetrathiotungstate precursor films much higher concentrations are required to achieve similar absorption levels to ammonium tetrathiomolybdate.

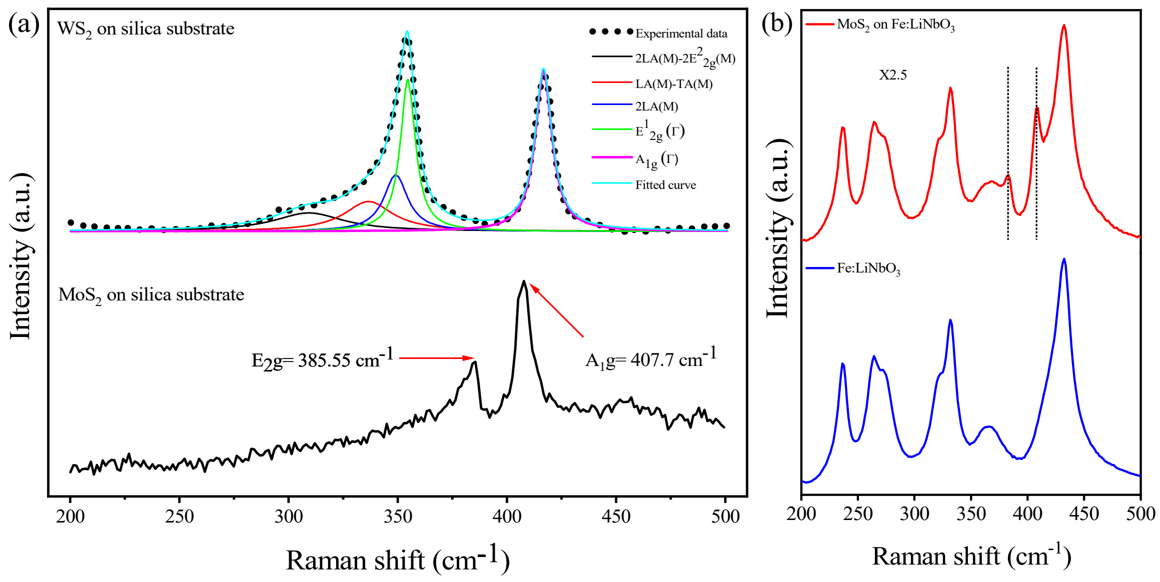

Figure S2: (a) Raman spectra of laser printed WS_2 (top) and MoS_2 (bottom) on pure silica substrate. The WS_2 Raman spectrum (using a pump laser at 532 nm) involves the convolved peak at around 350 cm^{-1} and A_1g at 417.6 cm^{-1}. The spectrum contains the constituent Raman lines corresponding to various vibrational modes of WS_2 which have been deduced by peak fitting. For WS_2, the peak intensity ratio $2LA/A_1g$ is 0.35 indicating that the WS_2 is bulk while the separation between Raman characteristics MoS_2 peaks A_1g and E_2g is $\simeq$22 cm^{-1} suggesting that MoS_2 is in the few-layer regime. (b) Raman spectra of laser deposited MoS_2 on lithium niobate (red curve) and bare lithium niobate substrate (blue curve). The dashed lines (in the red curve) are used to indicate the MoS_2 Raman peaks E_2g and A_1g which are at 383 cm^{-1} and 408 cm^{-1} respectively which corresponds to multilayer regime. The Raman modes labels of lithium niobate substrate can be found in Stone *et al.* [1].

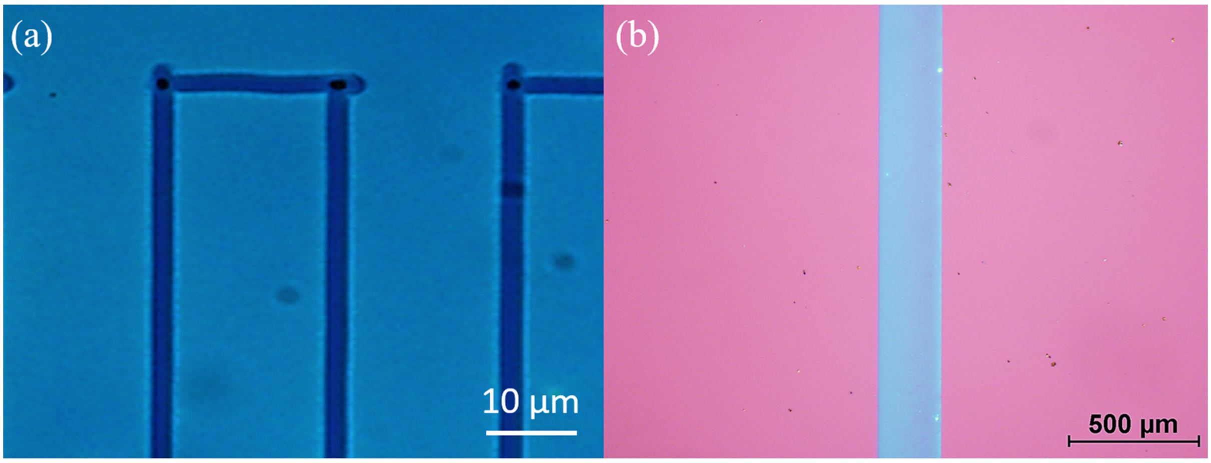

Figure S3: (a) Optical microscope image of laser written WS_2 tracks on a uniform $(NH_4)_2WS_4$ precursor film. (b) Optical micrograph of WS_2 film grown by overlapping raster scanning with 1cm length and 200 µm width (after developing in NMP to remove the unexposed part of the precursor films).

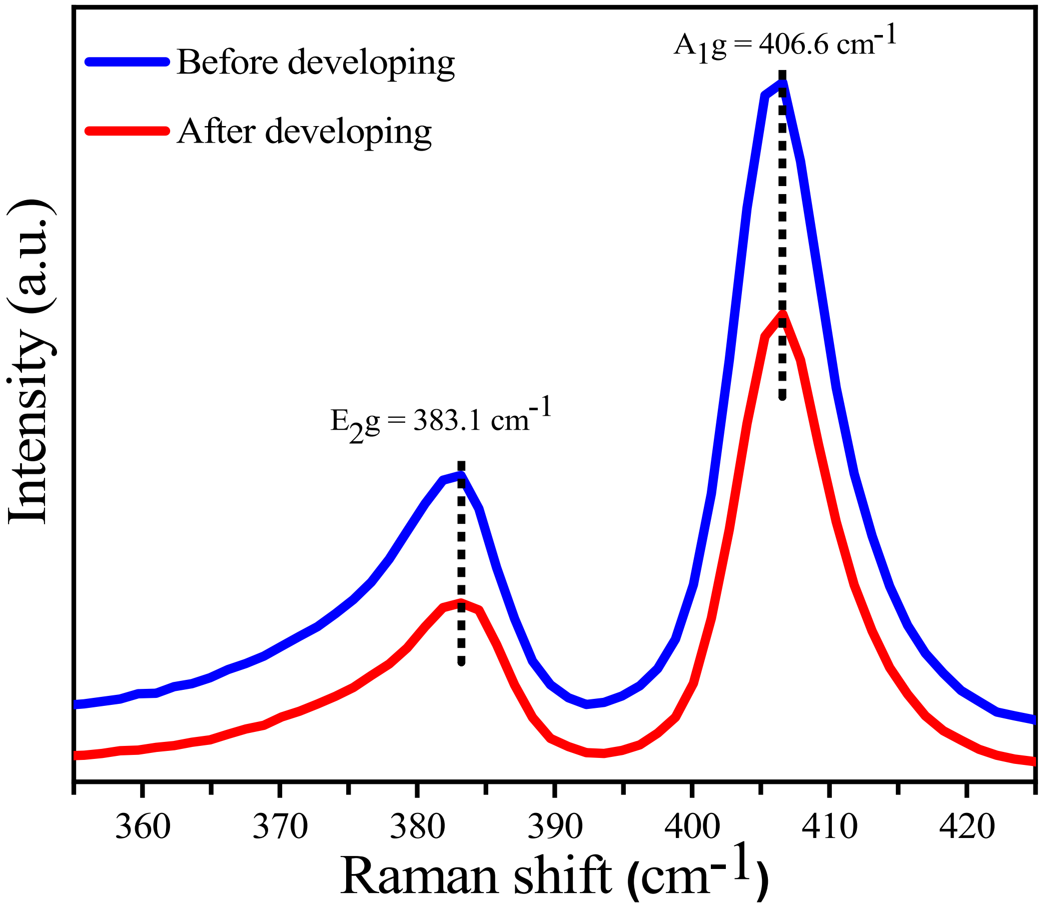

Figure S4: MoS_2 Raman peaks before and after development step in DMF solvent. There is no change in peak positions or FWHM of the peaks indicating that the development step in DMF does not alter the structure and composition of laser written MoS_2 films.

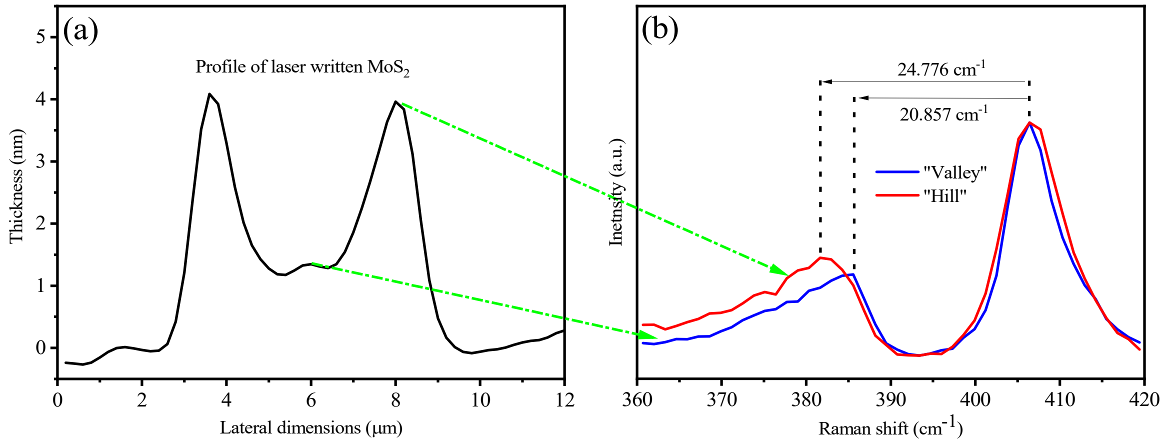

Figure S5: (a) Stylus profilometry measurement of single laser written MoS_2 track. Clearly, the central area of the MoS_2 track is thinner compared to the peripheral sides due to the Gaussian intensity distribution of the laser beam. (b) Comparison between MoS_2 Raman characteristics peaks positions A_1g and E_2g at the edges ("hills") in red trace and at the central area ("valley"). The separation in wavenumber between the two peaks is lower at the central area (20.857 cm^{-1}) while at the edge is larger (24.776 cm^{-1}) confirming the thickness of the MoS_2 at the centre is bilayer while at the edge is multilayer. Additionally, the Raman peaks FWHM for the "hills" appear broader due to exposure at lower laser intensity (i.e. lower temperature profile leads to lower crystallinity).

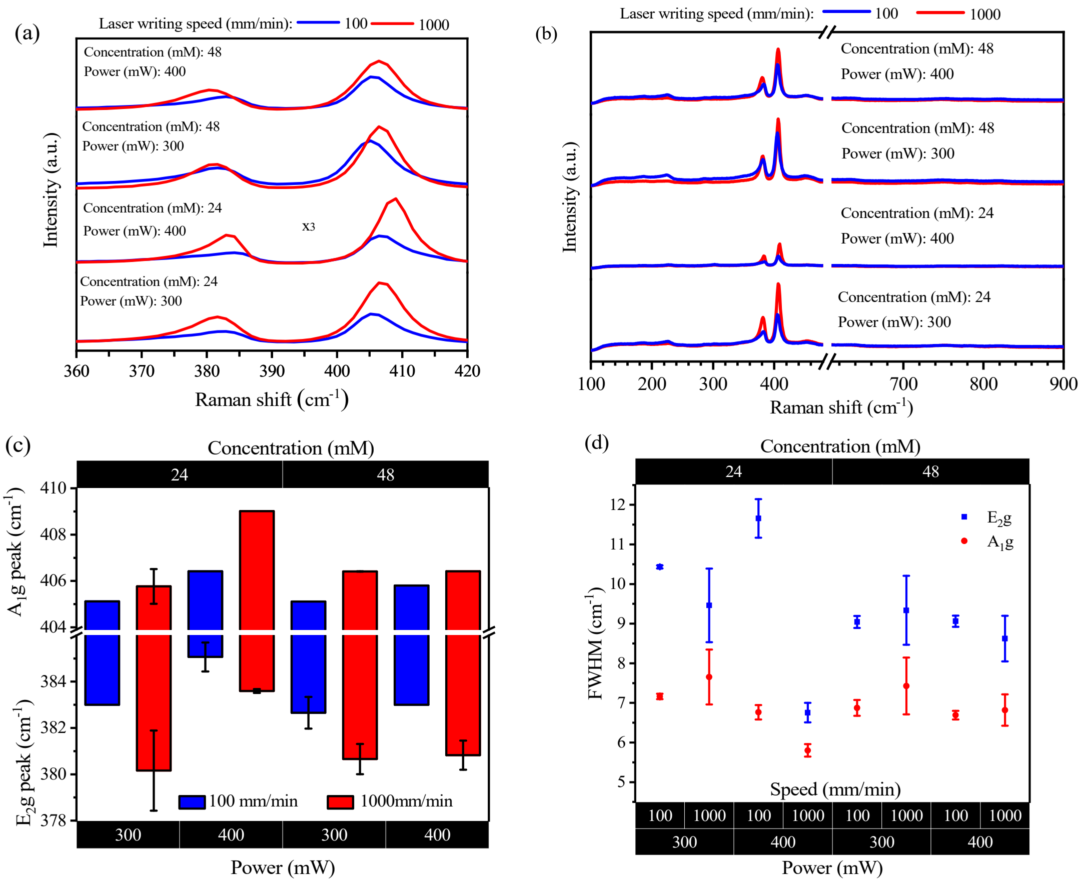

Figure S6: (a) Raman spectra showing the E_2g and A_1g Raman peaks corresponding to MoS_2 tracks synthesised using different laser irradiation conditions (using Ar^+ laser 514 nm and a 10x, 0.25 NA microscope objective). The A_1g - E_2g peak location difference, in wavenumbers, tends to be smaller at lower laser writing speeds indicating thinner MoS_2 films at these conditions. (b) Extended Raman spectra, ranging from 100 cm^{-1} to 900 cm^{-1}, of the same MoS_2 tracks at different laser irradiation conditions. No MoO_3 peaks were detected (normally they are located around 666 and 820 cm^{-1} [2,3]) regardless of the irradiation conditions. (c) Histogram of the average position of the active Raman modes (E_2g and A_1g) of MoS_2 films as a function of laser irradiation conditions. The error bars represent the standard deviation for four different tracks; No error bars mean that the Raman peaks in all four tracks have identical position. All E_2g peaks corresponding to laser scanning speed of 100 mm/min are shifted to higher wavenumber as compared with 1000 mm/min. Conversely, A_1g peaks are shifted to lower wavenumber due to thickness reduction (d) Graph represents the average FWHM of Raman active modes (E_2g and A_1g) of MoS_2 films as function of different writing conditions. Error bars

represent the standard deviation for four different tracks. Generally, the FWHM of A_1g peak for the laser-synthesised MoS_2 is 7±0.5 cm^{-1} regardless of the laser writing condition while FWHM of E_2g peak is reduced for thicker precursor films (48 mM) indicating improvement in crystallinity possibly due to higher absorption leading subsequently to higher peak temperatures within the irradiated spot.

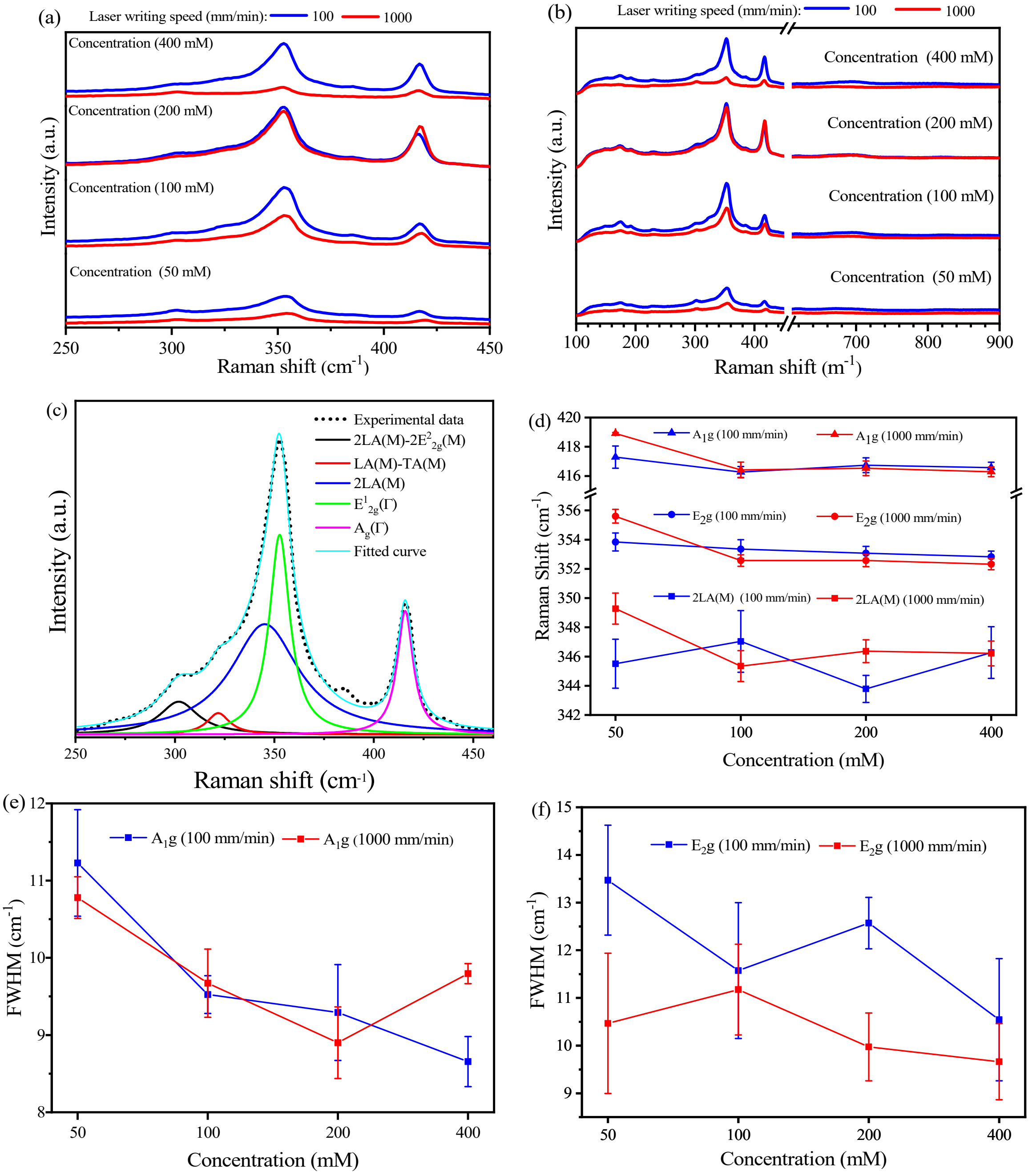

Figure S7: (a) Raman spectra of WS_2 tracks with convolution peak that includes E_2g with 2LA (M) and A_1g peaks at different scanning speeds and precursor concentrations (fixed laser power 300 mW with 20x, 0.4 NA microscope objective for focusing). (b) Extended Raman spectra of WS_2 films. A broad shallow shoulder around 700 cm^{-1} could indicate low WO_3 (WO_3 peaks are normally located at 710 cm^{-1} and 810 cm^{-1} [4,5]). (c) Lorentzian Peak deconvolution for WS_2 Raman spectra with 532 nm excitation wavelength. (d) Average positions of 2LA, E_2g and A_1g peaks as a function of different laser scanning

speeds and precursor concentrations. (e) The average FWHM of A_1g peak as a function of different laser scanning speeds and precursor concentrations. (f) The average FWHM of E_2g peak as a function of different laser scanning speeds and precursor concentrations. The error bars represent the standard deviation for five individual tracks.

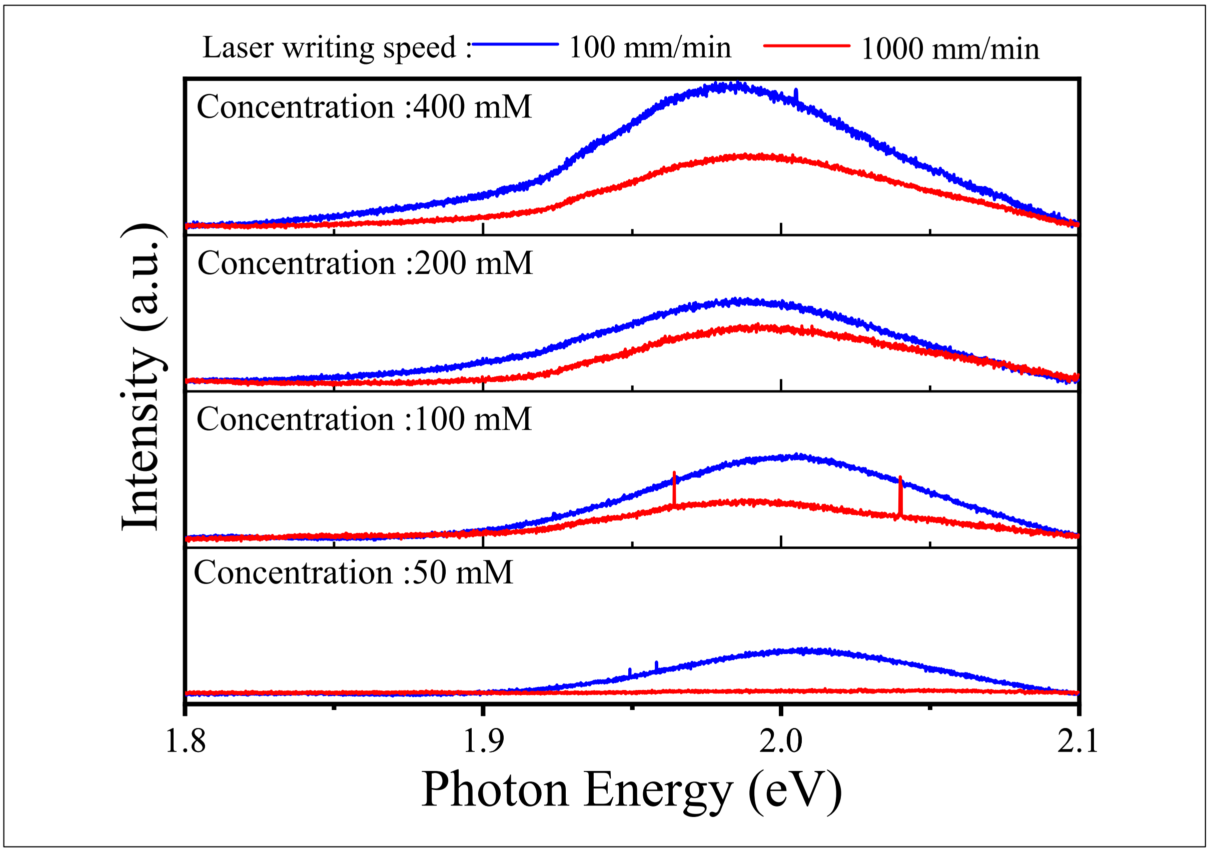

Figure S8: WS_2 PL peaks at different laser writing speeds for four different concentrations of the precursor.

A Renishaw inVia™ Raman Microscope with excitation wavelength 532 nm, focussed using a 50x objective, was used to study the room-temperature PL for both MoS_2 and WS_2 films. No PL was observed for MoS_2 films, irrespective of the laser excitation power level. In contrast, the WS_2 tracks, show clear PL peaks for all precursor concentrations due to its high quantum yield compared with MoS_2[6–8]. The PL peak shifts to higher energy, towards 2 eV, which is the energy of the direct band gap of WS_2 monolayers [6,9,10], when the concentration is reduced. At higher concentrations, 400 and 200 mM, the PL peak for 100 mm/min writing speed was located at 1.984 and 1.987 eV respectively whereas at 1000 mm/min the peaks are shifted to higher energies to be 1.99 eV. Similar PL peak positions has been reported in liquid exfoliated WS_2 flakes, which were laser etched to thicknesses of few layer [11]. At 100 mM precursor concentration, the trend of the laser writing speed was inverted where the peak at 100 mm/min is located at 2 eV which is assigned to (single-bilayer) and red-shifted

for 1000 mm/min to 1.992 eV. Finally, at the lowest concentration, 50 mM, the PL peak is also located at 2 eV for 100 mm/min speed while we could not find any peak located at this region for a scanning speed of 1000 mm/min. These results highlight the crucial effect of the initial precursor concentration and laser scanning speed (dwell time) on the total thickness of WS_2 tracks.

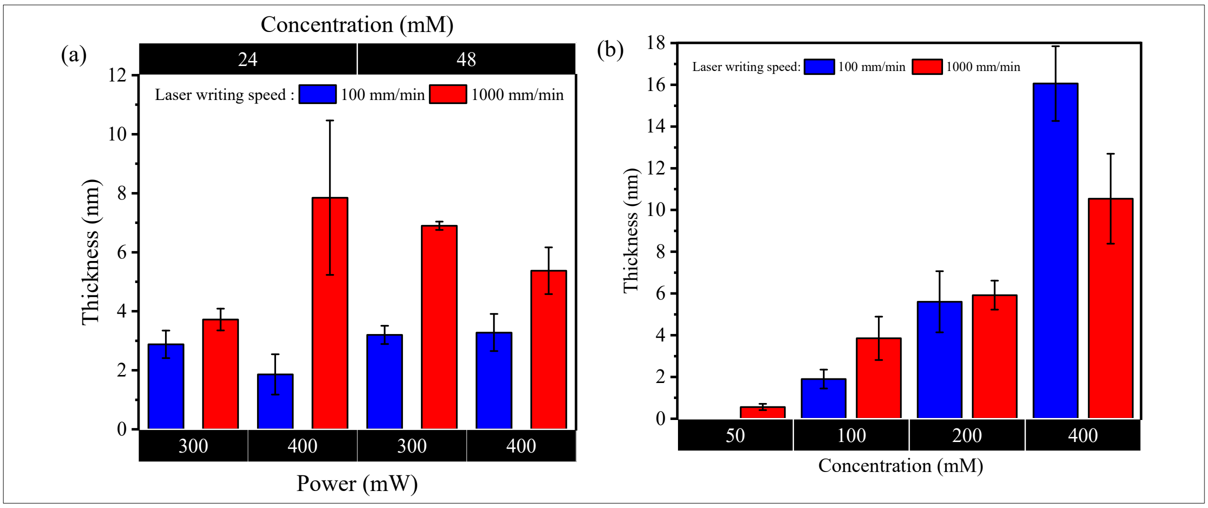

Figure S9: (a) Histogram of stylus profilometry measurement on MoS_2 tracks synthesised with different laser irradiation conditions and precursor concentrations. A 10x objective was used to focus the laser beam on the precursor film. (b) Histogram of stylus profilometry measurement on WS_2 tracks with different laser scanning speeds and precursor concentrations. The laser power is fixed at 300 mW. A 20x objective was used to focus the laser beam on the precursor film.

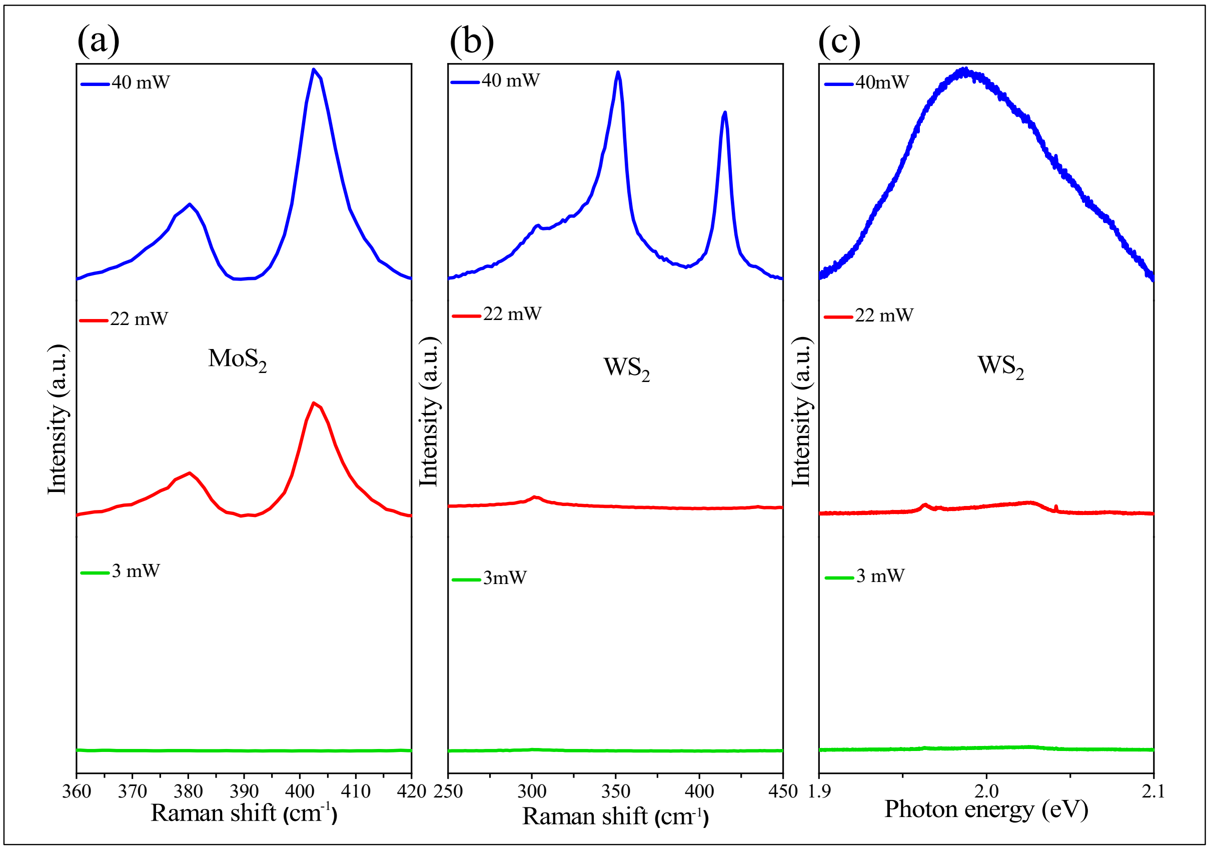

Figure S10: Real-time (*in-situ*) Raman spectra obtained simultaneously during laser synthesis of (a) MoS_2 and (b) WS_2 while (c) is PL spectra for WS_2 after laser formation (note that no PL signal corresponding to MoS_2 was detected). Power levels of 3, 22 and 40 mW were used with static exposure on 48mM precursor concentration films. At 3 mW, no MoS_2 or WS_2 peaks were observed. However, at 22 mW characteristic in-plane (380.3 cm^{-1}, E_2g) and out-of-plane (402.5 cm^{-1}, A_1g) peaks became pronounced, revealing rapid formation of MoS_2. No WS_2 formation was observed under the same irradiation conditions and precursor concentrations. However, at the maximum power of the pump laser beam, 40 mW, Raman peaks of MoS_2 (380.3 cm^{-1}, 402.5 cm^{-1}) and WS_2 (convoluted peak at 351.6 cm^{-1} and out-of-plane peak 415.6 cm^{-1}) emerged using both single-source precursors films. The intensity of MoS_2 peaks synthesised at 40 mW is higher compared to the MoS_2 peaks synthesised at 22 mW revealing an improvement in the crystallinity of the MoS_2 when it formed at higher laser power (corresponding to higher local temperature on the laser-irradiated spot).

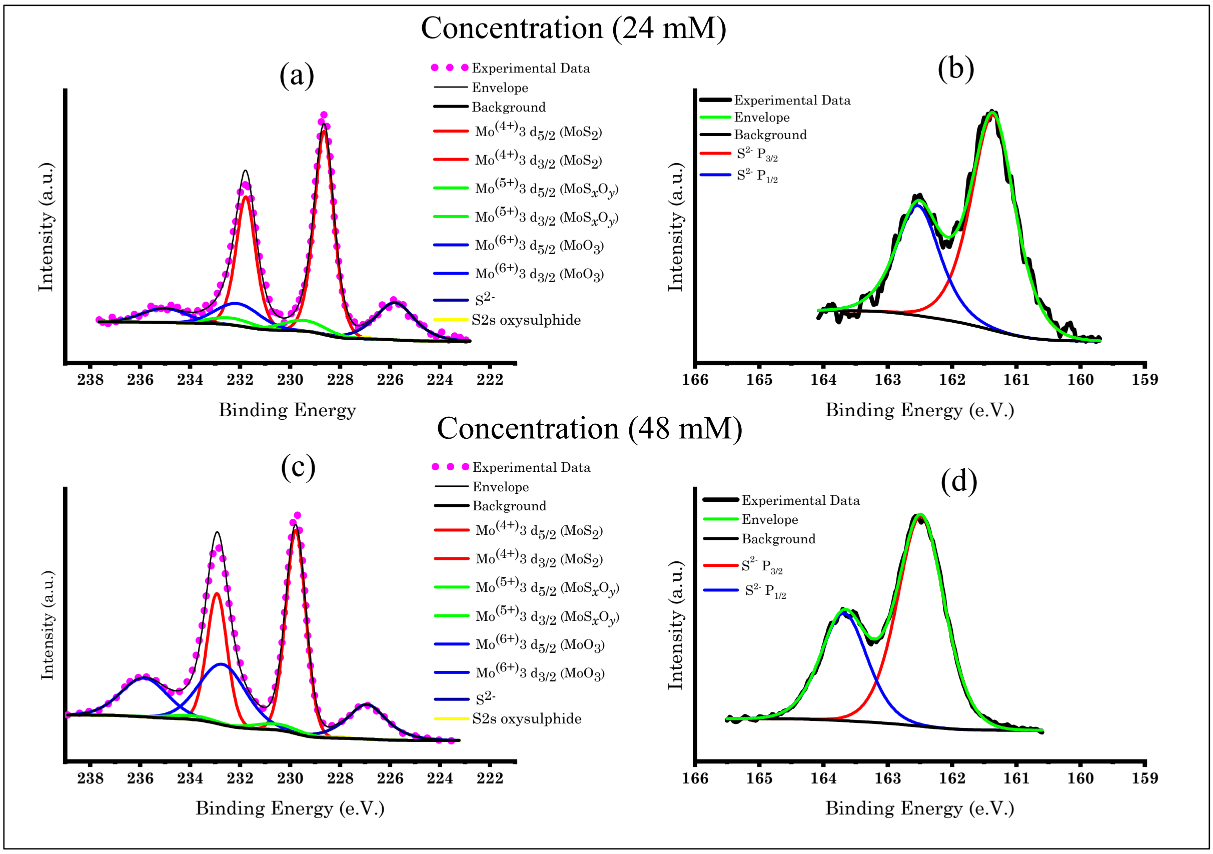

Figure S11: X-Ray photoelectron spectroscopy (XPS) spectra of laser written MoS_2 films using 24 and 48 mM precursor solution concentrations where (a) and (c) are Mo 3d core levels while (b) and (d) S 2p core levels.

Our direct laser writing protocol was performed under ambient conditions which means that relying on Raman data alone cannot give a complete picture of the microstructure stoichiometry and the effects of different growth conditions. XPS is an essential characterisation technique to study the composition of these TMDCs layers.

XPS was performed on 1 mm^2 area MoS_2 film samples generated by raster scanning an overlapping square pattern (the minimum area required for accurate XPS analysis). The first sample was made with 24 mM precursor film concentration and synthesised using 400 mW laser power and 100 mm/min writing speed, while the second sample was made with 48 mM precursor film, 300 mW power and 100 mm/min speed. The carbon peak C1s at 284.8 eV was used to calibrate the binding energy scale of XPS measurements for all TMDCs samples. For both samples, two core levels have been studied,

namely, the Mo 3d and the S 2p levels. For Mo core level, both 24 mM and 48 mM films exhibit three doublet and two singlet peaks while for the S 2p core, only one doublet was pronounced as shown in Fig S11. The first doublet $Mo^{(4+)}$ has two peaks labelled as $Mo^{(4+)}3\ d_{5/2}$ and $Mo^{(4+)}3\ d_{3/2}$ assigned to MoS_2 and it is located at 228.64 eV and 231.77 eV respectively for the 24 mM film while for 48 mM it shifted to higher energies at 229.77 eV and 232.92 eV respectively as can be seen in Fig S11 (a & c). A similar trend is observed in Fig. S11(b & d) for the S 2p core level where the doublet $S^{(2-)}$ with peaks labelled as $S^{(2-)}\ P_{3/2}$ and $S^{(2-)}\ P_{1/2}$ corresponding to MoS_2 are located at 161.38 eV and 162.56 eV respectively for the 24 mM film and are shifted to 162.49 eV and 163.69 eV for the 48 mM film. This blue shift of MoS_2 doublets in both cores at 48 mM film is attributed to higher temperature profile created by additional laser energy that is absorbed due to thicker $(NH_4)_2MoS_4$ films which decompose to more crystalline MoS_2 layers compared with 24 mM film [12]. This hypothesis is also supported by the analysis of FWHM of Raman peaks of MoS_2 (see Fig. S6(d)) where the films with higher concentrations have lower FWHM indicating higher crystallinity, i.e., they are annealed at higher temperature. Notably, the binding energies of MoS_2 synthesised by our approach with 48 mM precursor film are identical with MoS_2 synthesised by CVD approach at 900°C [13].The analysis of XPS data reveals that both MoS_2 films are sub-stoichiometric where the S/Mo atomic ratio is 1.73 for 24 mM and 1.77 for 48 mM. However, without an external source of sulphur, MoS_2 prepared by pyrolysis of $(NH_4)_2MoS_4$ is sulphur deficient with S/Mo atomic ratio 1.8 which is comparable to our findings [12].

Interestingly, the second $Mo^{(6+)}$ and third $Mo^{(5+)}$ doublets correspond to molybdenum oxide and molybdenum oxysulfide contents in our films, despite the fact that as shown in Fig. S6(b), these oxide species were not detected by Raman spectroscopy. This indicates they are substitution for sulphur vacancies in the MoS_2 lattice rather than individual molybdenum oxides layers [14]. The second doublet with peaks labelled $Mo^{(6+)}3\ d_{5/2}$ and $Mo^{(6+)}3\ d_{3/2}$, is assigned to MoO_3 formation and it is located at 232 eV and 235 eV respectively for 24 mM film whereas for 48 mM films, the peaks are found at 232.53 eV and 235.88 eV respectively. The last doublet is $Mo^{(5+)}3\ d_{5/2}$ and $Mo^{(5+)}3\ d_{3/2}$ which corresponds to

MoS_xO_y formation, located at 229.44 eV and 232.55 eV respectively for 24 mM film and 230.57 eV and 233.82 eV for the 48 mM film.

As is apparent from Fig. S11, the intensity of oxide peaks is increased proportionally with respect to the concentration (thickness) of the precursor films due to higher oxide content of MoS_2. To analyse this oxide content quantitatively, we calculate the oxide atomic ratio using the following formula [13]:- $\{(Mo^{(5+)}+Mo^{(6+)})/(Mo^{(4+)}+ Mo^{(5+)}+ Mo^{(6+)})\}$ for both films which is 25.7% for 24 mM and 38% for 48 mM film. The oxide content in our MoS_2 films is thus comparable for laser thinning of CVD MoS_2 [13] and is even lower for the 24 mM film. Clearly, the amount of oxide in our laser printed MoS_2 films depends heavily on the initial thickness (concentration) of the $(NH_4)_2MoS_4$ precursor layer compared to laser writing parameters.

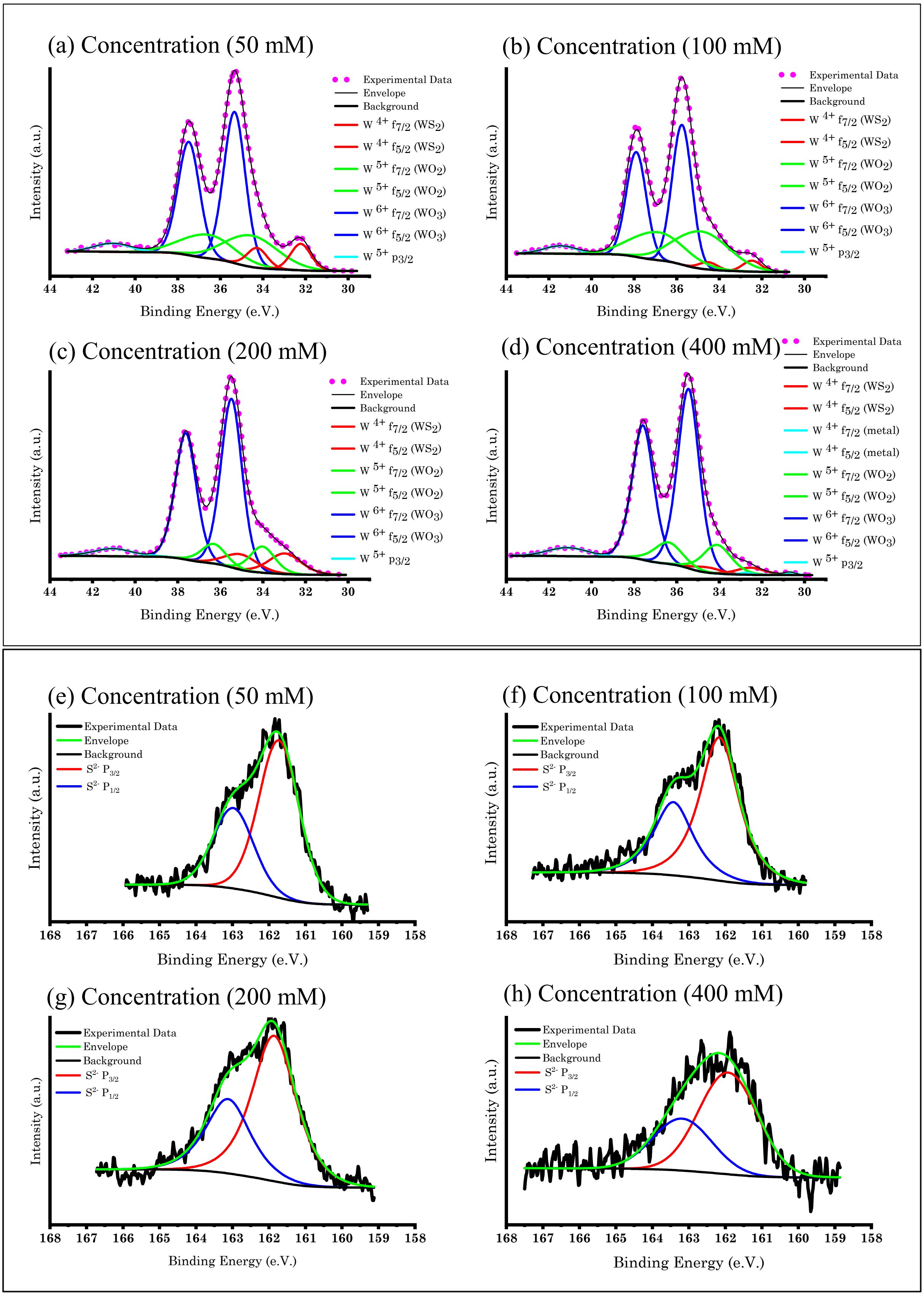

Figure S12: XPS spectra of laser written WS_2 films using 50, 100, 200 and 400 mM precursor solution concentrations where (a – d) show W 4f core levels and (e – h) show the S 2p core levels.

Four specimens of WS_2 with 1 mm^2 area (the minimum area required for accurate XPS analysis) synthesised by overlapping raster scanning has been tested with 50, 100, 200 and 400 mM precursor films concentrations with 300 mW power and 100 mm/min writing speed. Prior to deconvolution of XPS data for WS_2, the carbon peak $C1_S$ at 284.8 eV was used as a reference point to calibrate the XPS spectra.

To investigate the composition of WS_2, two core levels were studied, namely the W 4f and the S 2p core level. For W core level, two doublets are denoted in addition to one singlet for all concentrations, meanwhile one doublet is pronounced in the sulphur core level. Fig S12 depicts both cores respectively for all the concentrations that have been tested.

The first doublet $W^{(4+)}$ $f_{7/2}$ and $W^{(4+)}$ $f_{5/2}$ in W core level represents the formation of WS_2. The peak positions for this doublet is identical that reported previously for WS_2 flakes [15]. These are located at 32.25 eV and 34.25 eV for 50 mM film, 32.5 eV and 34.5 eV for 100 mM, 32.23 eV and 34 eV for 200 mM and 32.5 eV and 34.5 eV for 400 mM film.

Simultaneously, in S 2p core level, the doublet (S^{2-} $P_{3/2}$ and S^{2-} $P_{1/2}$) that corresponds to WS_2 is located at: 161.77 eV and 163 eV for 50 mM, 162.1 eV and 163.45 eV for 100 mM, 161.8 eV and 163 eV for 200 mM, and 161.6 eV and 162.8 eV for 400 mM film. Consequently, the S/W atomic ratio has been calculated to be: 1.84 for 50 mM film, 1.83 for 100 mM film, 2.26 for 200 mM film and finally 1.31 for 400 mM film. Although the stoichiometry for the first two films is slightly sulphur deficient while the third film is over sulphurized, they still in the acceptable composition margin of WS_2. However, we anticipate that further optimisation for the laser writing conditions, in particular, for the precursor film concentrations could results the desired S/W atomic ratio of 2.

The second doublet $W^{(6+)}$ $f_{7/2}$ and $W^{(6+)}$ $f_{5/2}$ in W core level corresponds to WO_3 formation and the binding energies for this doublet is identical to that reported previously [16,17] for WO_3. These are located at: 35.35 eV and 37.46 eV for 50 mM film, 35.8 eV and 37.8 eV for 100 mM film, 35.5 eV and

37.6 eV for 200 mM film, and 35.5 eV and 37.6 eV for 400 mM film. Moreover, the intensity of second doublet peaks in W core level ($W^{(6+)}$ $f_{7/2}$ and $W^{(6+)}$ $f_{5/2}$) which is assigned to the WO_3 formation, significantly overwhelmed the first doublet of WS_2 indicating that the oxide content is much higher compared to laser printed MoS_2 films prepared by overlapping raster scanning (see Fig. S11). This could be attributed to the nature of oxidation process of WS_2 which is photo-induced effect [18]. However, the oxidisation level is still proportional to the precursor film thickness as in the MoS_2 case. Nevertheless, as Raman spectroscopy did not reveal any obvious vibrational modes for the WO_3 (see Fig. S7(b)), this indicates, just as with the MoS_2 layers, that the films contain oxidised WS_2 rather than two individual layers of WS_2 and WO_3 (but with a higher level of oxidation for WS_2 compared to MoS_2) [14]. Compared to XPS literature reports for laser thinned WS_2[19–22], our laser printed WS_2 layers exhibit a comparable amount of oxide.

In conclusion, the XPS analysis of TMDCs films, prepared by overlapping raster scanning (as detailed in Fig. S11 and Fig. S12), suggests that the oxide content in both MoS_2 and WS_2 films is overestimated compared to individual laser synthesized tracks. We hypothesise that the overlapping raster scanned films are necessarily exposed to laser power for long durations under ambient conditions, i.e. exposed to annealing at high temperature for longer time in air causing an increase in the oxide content of TMDCs films.

Furthermore, the high absorption of laser energy by the thicker precursor films creates a steep temperature profile under ambient operating conditions, thus giving rise to increased oxide formation. Notably, this property is also characteristic of laser post-treated TMDCs flakes where the amount of oxide is increased when the flakes are thicker comparing to the thinned ones [11,23,24].

Finally, from the results of Raman, PL and stylus profilometry of laser printed TMDCs single tracks as well as the XPS analysis of TMDCs films formed by raster-scanning, we can anticipate that using lower concentration precursor films can form thinner TMDCs with lower oxide contents but lower crystallinity and vice versa when high concentration precursor films are used for laser printing.

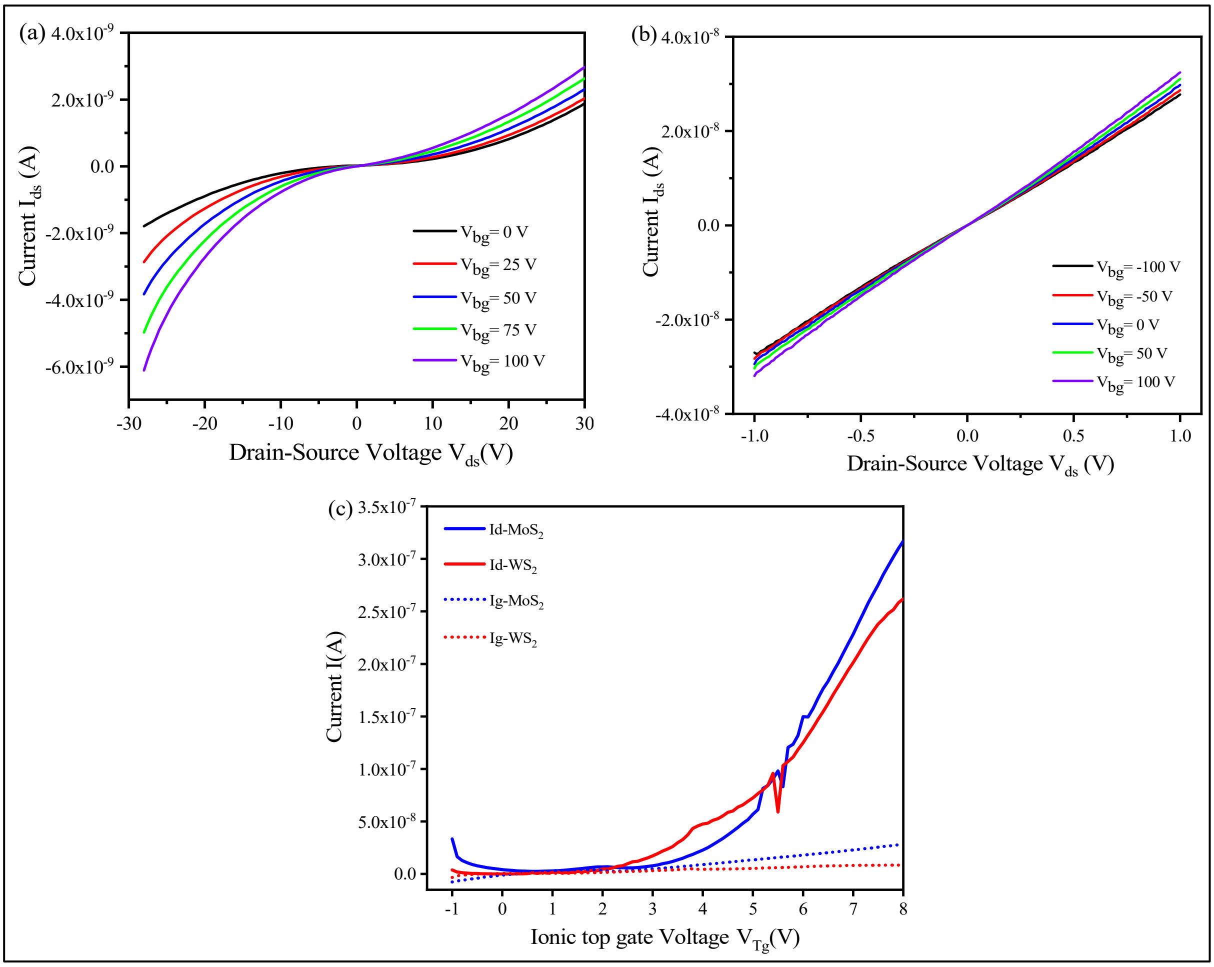

Figure S13: Output characteristics of backgated (a) MoS_2 FET and (b) WS_2 FET. (c) Linear scale transfer characteristics of MoS_2 and WS_2 with top ionic gated FET configuration.

The output characteristics of back gated devices reach the minimum output current at zero drain-source voltage regardless of the voltage applied to the gate indicating low leakage gate current. The output characteristics of MoS_2 FET device has a nonlinear Schottky behaviour while the WS_2 device possesses an ohmic linear resistance.

The field effect mobility was extracted from the slope of the linear part of the transfer curve using the equation:

$$\mu_{FE} = \frac{L}{W\ C_{OX}\ V_{ds}} \frac{\Delta I}{\Delta V_g}$$

Where μ_{FE} is the field effect mobility, L is the channel length, *W* is the channel width, Vds is the source-drain voltage and ($\Delta I/ \Delta Vg$) is the transconductance. C_{ox} is the gate capacitance per unit area of silicon dioxide layer (290 nm) which is (12 nF/cm^2) for backgated devices while the gate capacitance per unit area for ionic top-gated devices formed by poly (ethylene oxide) (PEO) and lithium perchlorate ($LiClO_4$) is estimated to be 1 µF/cm^2 as stated by Lin et al. [25]. The ionic gel recipe and preparation has been adopted with trivial modifications from Hu et al. [26]. These values are comparable to MoS_2 FETs with the same configuration that were also fabricated using single source precursors [27,28], but where the TMDCs channels were patterned by conventional top-down lithography.

Table 1: TMDCs device parameters:

Parameter	MoS_2-Back gate	MoS_2-Ionic gate	WS_2- Back gate	WS_2-Ionic gate
Threshold voltage (V)	-50.5	4.45	-	4.33
I(min) nA	2.0225	2.5	34.5	$3x10^{-2}$
I(max) nA	6.0225	317	41.2	261.8
on-off ratio	2.978	127	1.1942	8728
Transconductance (S)	$3.94x10^{-11}$	$8.842x10^{-8}$	$3.4x10^{-11}$	$7.442x10^{-8}$
Capacitance (F/cm^{-2})	$1.2x10^{-8}$	10^{-6}	$1.2x10^{-8}$	10^{-6}
Mobility (cm^2/V.S)	$5.51x10^{-5}$	$7x10^{-3}$	$1.142x10^{-4}$	$6 x10^{-3}$

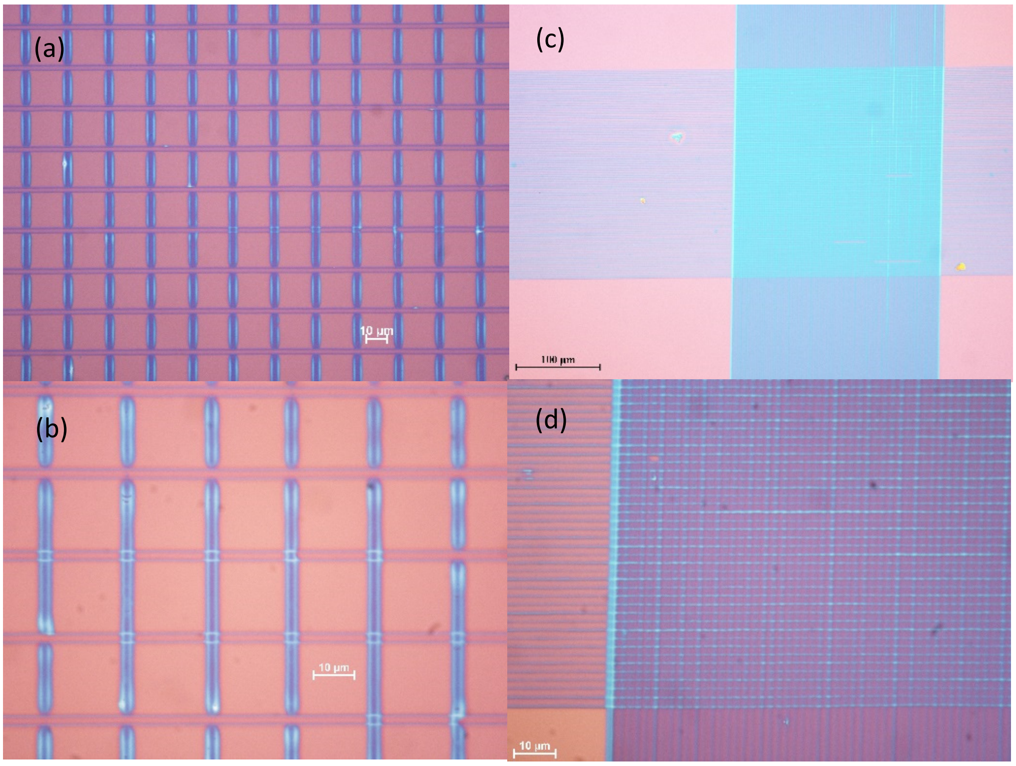

Figure S14: (a) Optical microscopy image of an array of WS_2/MoS_2 heterostructures. Although numerous junctions have been created successfully, some are damaged due to the non-optimised synthesis parameters as can be seen at higher resolution in (b). (c) Optical microscopy image of an array of multiple overlapping WS_2/MoS_2 tracks forming a large area heterojunction. (d) High resolution optical micrograph showing "tartan" pattern of crosshatched, densely packed, overlapping WS_2/MoS_2 lines.

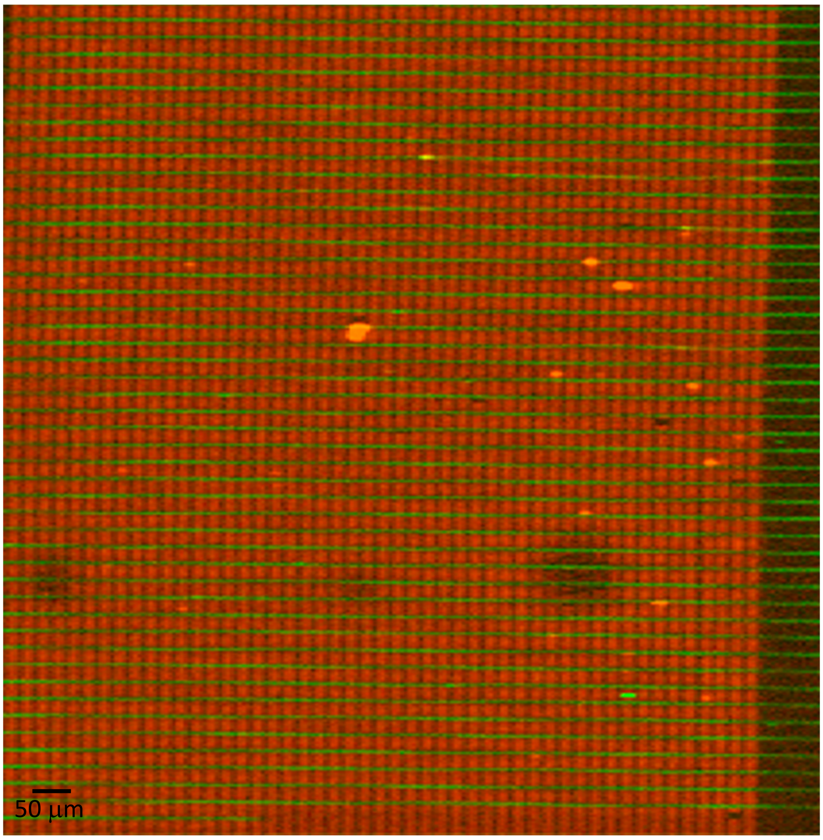

Figure S15: False colour X-Ray fluorescence (XRF) maps corresponding to the X-Ray emission of Mo and W atoms in a laser printed, 50 x 50 MoS_2/WS_2 heterojunction array (50 lines/mm). These maps were obtained at the micro XRD beam line (I18) at the Diamond light source, Harwell campus, Oxfordshire, UK. The energy of the excitation beam was 20.1 keV. The sample was scanned in front of the focussed X-Ray excitation beam and a spectrum was obtained at each point, every 5 μm on the horizontal direction and every 1 μm on the vertical. This is due to the asymmetry of the excitation beam. An energy dispersive X-Ray detector was used to monitor the Kα emission of Mo (red tracks) and Lα emission for W (green tracks) thus indicating the spatial distribution of those elements on the sample.

References:

1. Stone, G., Knorr, B., Gopalan, V. & Dierolf, V. Frequency shift of Raman modes due to an applied electric field and domain inversion in $LiNbO_3$. *Phys. Rev. B* **84**, 134303 (2011).

2. Windom, B. C., Sawyer, W. G. & Hahn, D. W. A raman spectroscopic study of MoS_2 and MoO_3: Applications to tribological systems. *Tribol. Lett.* **42**, 301–310 (2011).

3. Wang, T., Li, J. & Zhao, G. Synthesis of MoS_2 and MoO_3 hierarchical nanostructures using a single-source molecular precursor. *Powder Technol.* **253**, 347–351 (2014).

4. Gutiérrez, H. R. *et al.* Extraordinary room-temperature photoluminescence in triangular WS_2 monolayers. *Nano Lett.* **13**, 3447–3454 (2013).

5. Chen, T. Y. *et al.* Comparative study on MoS_2 and WS_2 for electrocatalytic water splitting. *Int. J. Hydrogen Energy* **38**, 12302–12309 (2013).

6. Yuan, L. & Huang, L. Exciton dynamics and annihilation in WS_2 2D semiconductors. *Nanoscale* **7**, 7402–7408 (2015).

7. Zhao, W. *et al.* Evolution of Electronic Structure in Atomically Thin Sheets of WS_2 and WSe_2. *ACS Nano* **7**, 791–797 (2013).

8. Peimyoo, N. *et al.* Nonblinking, Intense Two-Dimensional Light Emitter: Monolayer WS_2 Triangles. *ACS Nano* **7**, 10985–10994 (2013).

9. Elías, A. L. *et al.* Controlled synthesis and transfer of large-area WS_2 sheets: From single layer to few layers. *ACS Nano* **7**, 5235–5242 (2013).

10. Xu, Z. Q. *et al.* Synthesis and Transfer of Large-Area Monolayer WS_2 Crystals: Moving Toward the Recyclable Use of Sapphire Substrates. *ACS Nano* **9**, 6178–6187 (2015).

11. Bissett, M. A., Hattle, A. G., Marsden, A. J., Kinloch, I. A. & Dryfe, R. A. W. Enhanced Photoluminescence of Solution-Exfoliated Transition Metal Dichalcogenides by Laser Etching. *ACS Omega* **2**, 738–745 (2017).

12. Sygellou, L. An in-situ photoelectron spectroscopy study of the thermal processing of ammonium tetrathiomolybdate, $(NH_4)_2MoS_4$, precursor. *Appl. Surf. Sci.* **476**, 1079–1085 (2019).

13. Lu, J. *et al.* Improved Photoelectrical Properties of MoS_2 Films after Laser Micromachining. *ACS Nano* **8**, 6334–6343 (2014).

14. Sirota, B., Glavin, N. & Voevodin, A. A. Room temperature magnetron sputtering and laser annealing of ultrathin MoS_2 for flexible transistors. *Vacuum* **160**, 133–138 (2019).

15. Voiry, D. *et al.* Enhanced catalytic activity in strained chemically exfoliated WS_2 nanosheets for hydrogen evolution. *Nat. Mater.* **12**, 850–855 (2013).

16. Sun, Y. *et al.* Photoelectrochemical and structural characterization of carbon-doped WO_3 films prepared via spray pyrolysis. *Int. J. Hydrogen Energy* **34**, 8476–8484 (2009).

17. Benoit, A., Paramasivam, I., Nah, Y. C., Roy, P. & Schmuki, P. Decoration of TiO_2 nanotube layers with WO_3 nanocrystals for high-electrochromic activity. *Electrochem. commun.* **11**, 728–732 (2009).

18. Kotsakidis, J. C. *et al.* Oxidation of Monolayer WS_2 in Ambient Is a Photoinduced Process. *Nano Lett.* **19**, 5205–5215 (2019).

19. Huang, C. C. *et al.* Transfer-Free Growth of Atomically Thin Transition Metal Disulfides Using a Solution Precursor by a Laser Irradiation Process and Their Application in Low-Power Photodetectors. *Nano Lett.* **16**, 2463–2470 (2016).

20. Venkatakrishnan, A. *et al.* Microsteganography on WS_2 Monolayers Tailored by Direct Laser Painting. *ACS Nano* **11**, 713–720 (2017).

21. Ma, S. *et al.* Enhanced Photocatalytic Activity of WS_2 Film by Laser Drilling to Produce Porous WS_2/WO_3 Heterostructure. *Sci. Rep.* **7**, 3125 (2017).

22. Atkin, P. *et al.* Laser exposure induced alteration of WS_2 monolayers in the presence of ambient moisture. *2D Mater.* **5**, 15013 (2017).

23. Alrasheed, A. *et al.* Surface Properties of Laser-Treated Molybdenum Disulfide Nanosheets for Optoelectronic Applications. *ACS Appl. Mater. Interfaces* **10**, 18104–18112 (2018).

24. Tran Khac, B. C. *et al.* Laser-Induced Particle Adsorption on Atomically Thin MoS_2. *ACS Appl. Mater. Interfaces* **8**, 2974–2984 (2016).

25. Lin, M.-W. *et al.* Mobility enhancement and highly efficient gating of monolayer MoS_2 transistors with polymer electrolyte. *J. Phys. D. Appl. Phys.* **45**, 345102 (2012).

26. Hu, H. *et al.* Broadly tunable graphene plasmons using an ion-gel top gate with low control voltage. *Nanoscale* **7**, 19493–19500 (2015).

27. Lim, Y. R. *et al.* Roll-to-Roll Production of Layer-Controlled Molybdenum Disulfide: A Platform for 2D Semiconductor-Based Industrial Applications. *Adv. Mater.* **30**, 1705270 (2018).

28. Lim, Y. R. *et al.* Wafer-Scale, Homogeneous MoS_2 Layers on Plastic Substrates for Flexible Visible-Light Photodetectors. *Adv. Mater.* **28**, 5025–5030 (2016).
